# Vascular anatomy is a determining factor of successful submental flap raising: a retrospective study of 70 clinical cases

**DOI:** 10.7717/peerj.3606

**Published:** 2017-09-19

**Authors:** Hung-Che Lin, Yuahn-Sieh Huang, Yueng-Hsiang Chu, Shao-Cheng Liu, Wei-Chuan Shangkuan, Wen-Sen Lai, Jinn-Moon Yang, Yaoh-Shiang Lin, Kuo-Hsing Ma, Jih-Chin Lee

**Affiliations:** 1 Department of Otolaryngology—Head and Neck Surgery, Tri-Service General Hospital, National Defense Medical Center, Taipei, Taiwan; 2 Department of Biology and Anatomy, National Defense Medical Center, Taipei, Taiwan; 3 National Defense Medical Center, Taipei, Taiwan; 4 Department of Otolaryngology—Head and Neck Surgery, Taichung Armed Forces General Hospital, Taichung, Taiwan; 5 Department of Biological Science and Technology, Institute of Bioinformatics and Systems Biology, National Chiao Tung University, Hsinchu, Taiwan; 6 Department of Otolaryngology Head and Neck Surgery, Kaohsiung Veterans General Hospital, Kaohsiung, Taiwan

**Keywords:** Venous return, Submental flap, Head and neck cancer, Reconstruction

## Abstract

The vascular anatomy of submental flaps (SFs) represents a determining factor in successful SF raising. However, little attention has been focused on the venous return of SFs. Thus, the present study aimed to investigate SF venous return. This study enrolled patients who underwent SF reconstructive surgery in a tertiary referral center between November 2009 and October 2016. The drainage pathway of the SF venous return was routinely identified during the course of our operations to prevent damage during head and neck surgery. The venous return data of 70 patients were reviewed. The size of the flaps ranged from 15 to 84 cm^2^, and total flap loss was not observed in the case series. All of the submental arteries originated from the facial artery; however, the submental veins of 70 patients returned to either the internal jugular vein (IJV, 72.9%) or the external jugular vein (EJV, 27.1%). Our data suggest that drainage of the submental vein into the EJV, which has been previously overlooked, should receive greater attention during SF surgeries. The results support mandatory preservation of the EJV and IJV and indicate that vascular anatomy is a determining factor for successful SF raising.

## Introduction

Free flaps constitute the most common method of reconstructing orofacial defects caused by head and neck cancer surgery. Free forearm flaps and anterior lateral thigh free flaps are two popular flap types; however, donor-site morbidity is frequently observed, and it has a considerable effect on the patient’s quality of life. Moreover, the flap design and surgical technique require longer operation times and the involvement of another microsurgical team. Such surgeries also present challenges that arise from the potential failure of vascular anastomosis and higher costs (Miller et al., 2015). Moreover, cosmetic skin color matches are difficult to achieve between free flaps and facial skin.

However, submental flaps (SFs) represent an easier and more efficient method of generating pedicled flaps for head and neck reconstruction. The application of SFs introduces less donor-site morbidity and enables reconstruction in one stage (Lee et al., 2013a; Lee et al., 2013b). Generally, reconstruction with SFs can be accomplished during neck dissection within one hour. Furthermore, the scar at the donor site can be easily hidden behind the mandibular arch. Many recent studies have demonstrated that SFs are suitable for reconstructing small and medium-sized defects in the head and neck region (Babu, 2012; Magden et al., 2004; Martin et al., 1993; Pinar, Govsa & Bilge, 2005; Pistre et al., 2001). Forner et al. (2016) also showed that the reconstruction of oral cancer defects with SFs was less costly than radial forearm free flaps. Additionally, free flap surgery requires specialized expertise that may not be readily available in all centers. Furthermore, this technique is associated with longer operative times and more extensive postoperative monitoring in intensive care units (Kokot et al., 2013). Because we do not need to consult a plastic surgeon for reconstruction, this operation can be scheduled five days per week.

SF application was first reported by Martin et al. (1993) using 20 fresh cadavers and eight patients who underwent orofacial reconstruction. The anatomy was described via clinical applications, and surgical guidelines were provided. Because vasculature is critical for the survival of pedicled SFs, detailed anatomies of the facial artery, the submental artery, and the submental vein have been described in a number of earlier studies. For example, Magden et al. (2004) reported the locations and dimensions of the facial and submental arteries based on 13 formalin-preserved cadavers. Pinar, Govsa & Bilge (2005) presented detailed anatomical descriptions of the submental artery derived from studies of 25 formalin-preserved cadavers. However, anatomical variations that occur after the facial vein have not been previously described in detail.

Pistre et al. (2001) were the first authors to discuss the anatomy of the facial vein and the submental vein in detail, and they provided a general understanding of the submental venous return. Based on their study, the submental vein was found to drain into the facial vein, where it eventually formed the common facial vein, which returned exclusively to the internal jugular vein (IJV). Draining of the common facial vein directly into the external jugular vein (EJV) was not reported until 2012 in a case report of one adult male cadaver (Babu, 2012).

Because the various drainage patterns of the veins are essential to flap design and surgical techniques, the aim of our work was to conduct an anatomical study of the venous return of the SF in 70 live surgeries (Magden et al., 2004; Martin et al., 1993; Pinar, Govsa & Bilge, 2005; Pistre et al., 2001).

## Materials and Methods

### Design

This was a retrospective study.

### Setting

This study was conducted at the Tri-Service General Hospital, Taipei, Taiwan between November 2009 and October 2016.

### Ethical considerations

Retrospective reviews of medical charts were approved by the Ethics Committee of the Tri-Service General Hospital, Taiwan. The study protocol was approved by the Institutional Review Board of Tri-Service General Hospital (TSGH IRB No. 1-105-05-026).

### Participants

A total of 70 head and neck cancer patients (63 male, 7 female) aged 32–89 years (mean age: 57.06 ± 12.73 years) received surgical treatment via SF reconstruction in the Tri-Service General Hospital of Taipei, Taiwan from November 2009 to October 2016.

### Data sources

Personal and medical histories were collected from all of the patients. Data on the participants’ demographic characteristics (age and sex), age at initial treatment, clinical diagnosis, cancer stage, flap size, total or partial flap loss, anatomy of venous returns, adjuvant therapy methods, tumor recurrence (locoregional metastasis, nodal metastasis, distant metastasis, etc.), survival status, occult metastases, and follow-up length were analyzed.

### Surgical method

**Figure 1:**
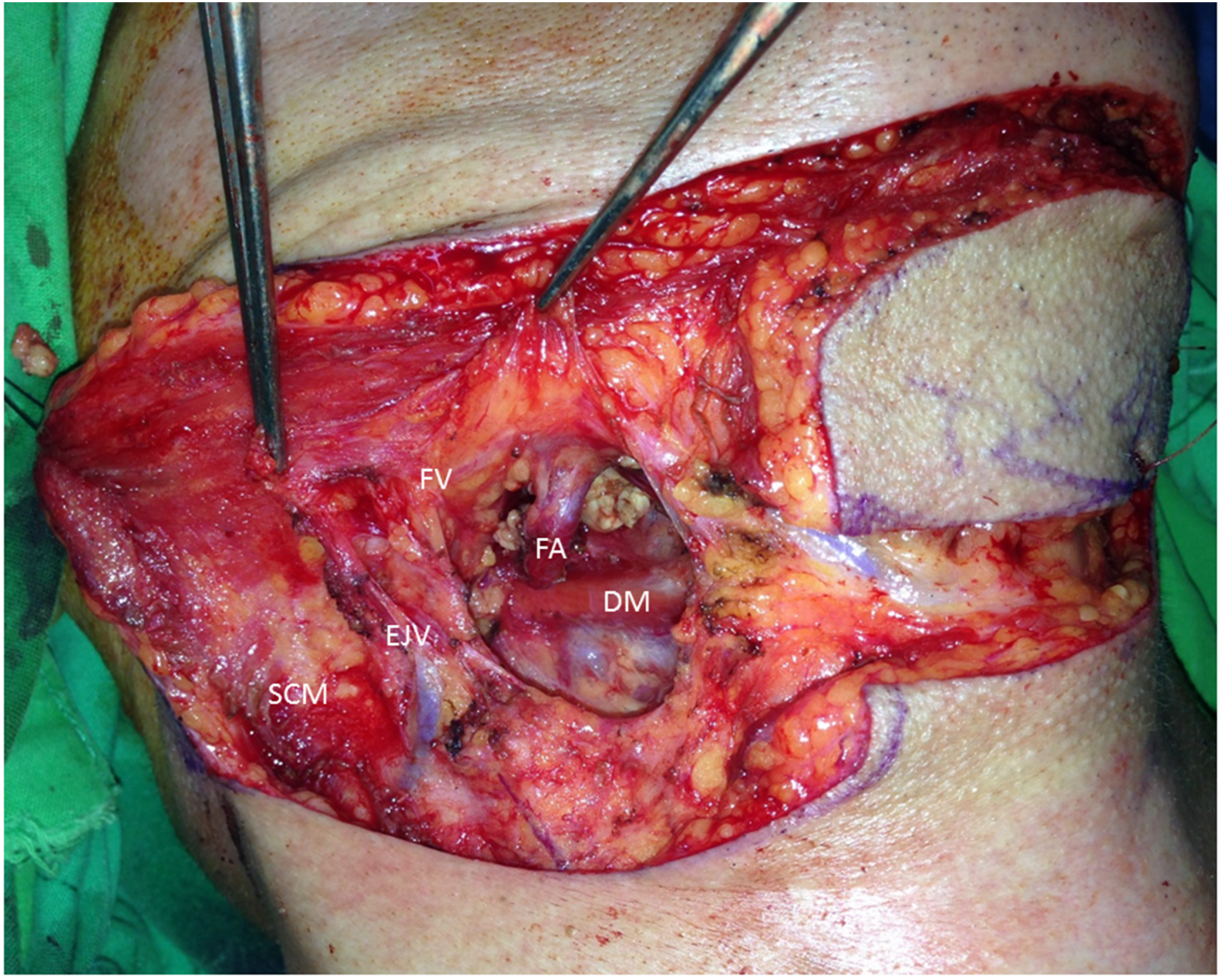
Facial vein drainage to the EJV. After removing the submandibular gland, the feeding artery (FA: facial artery) and drainage vein (FV: facial vein) can be clearly observed. In this case, the facial vein drains to the EJV. The sternocleidomastoid muscle (SCM) and digastric muscle (DM) are annotated.

The surgical method started with the flap design. The flap was dissected by removing the submandibular gland, and the vasculature of the flap, including the feeding artery and drainage vein, were clearly observed (Fig. 1). The facial vein should be preserved, and the facial artery should not be damaged. The drainage pathway of the SF arterial and venous return was routinely identified in the course of our operations to prevent damage during head and neck surgery.

Two criteria had to be met to utilize the SF: a clear neck situation with N0 status as observed preoperatively via a clinical examination and regional MRI and an acceptable defect size as assessed preoperatively via a clinical examination, regional MRI and intraoperative assessment. According to the original report by Martin et al. (1993), the defect size should be smaller than 126 cm^2^ (18 × 7 cm) for primary closure of the submental donor-site skin.

### Statistical methods

IBM SPSS Statistics software version 22 (IBM, Armonk, NY, USA) was used for the statistical analysis. Demographic and clinical characteristics are described using the mean values, standard deviations, and percentages.

## Results

A total of 70 patients consisting of 63 men (90%) and seven women (10%) were included. The mean age was 57.06 ± 12.73 years (range, 32–89). All of the surgical procedures were performed by the same surgeon (JC Lee), and the mean follow-up period was 36.69 ± 14.32 months. The flap sizes ranged from 5 × 3 cm to 12 × 7 cm, and 66 patients underwent combined neck dissection. All of the flaps survived, although three cases encountered partial marginal necrosis without requiring additional surgery. Most of the patients received neck dissection (94.3%) and adjuvant therapy (71.4%). In total, 20 patients (28.6%) were treated by surgery alone, 13 (18.6%) were treated by surgery accompanied by radiotherapy, and 37 (52.9%) received surgery in conjunction with concurrent chemoradiotherapy (CCRT). More than half of the patients were classified as N0 (38 patients, 54.3%), followed by N1 (17 patients, 24.3%), N2b (9 patients, 12.9%) and N2c (1 patient, 1.4%). The distribution of occult metastatic lymph nodes most frequently involved level II, III, and IV (*n* = 14, 20.0%) and then level I (*n* = 15, 15.7%). Favorable outcomes occurred in 61 (87.1%) surviving patients. However, nine patients died of the disease. The clinical and oncological data are shown in Tables 1 and 2. Cancer was most commonly located in the buccal mucosa, followed by the tongue, hypopharynx and mouth floor.

**Table 1:** Clinical and oncological results of patients treated with SF.

Variables	Values
Age (y)	57.06 ± 12.73
Gender	
Male	63
Female	7
Flap size	15–84
Primary subsite	
Tongue	20
Floor of mouth	8
Buccal mucosa	21
Lip	2
Soft palate	1
Hard palate and soft palate	1
Gingiva	5
Hypopharynx and larynx	10
Others	2
Level of neck dissection performed	
None	4
I–III	41
I–IV	14
I–V	11
Adjuvant therapy	
Radiotherapy	14
Concurrent chemoradiotherapy (CCRT)	38
None	20
Flap loss	
Partial	3
Total	0
Survival	
Yes	61
No	9

**Table 2:** Pathological staging and recurrence patterns.

Variables	Frequency	Percent
T staging		
T1	15	21.4%
T2	31	44.3%
T3	9	12.9%
T4	15	21.4%
Histology		
Squamous cell carcinoma (SCC)	67	95.7%
Non-SCC	3	4.3%
Differentiation		
Well differentiated	18	25.7%
Moderately differentiated	39	55.7%
Poorly differentiated	9	12.9%
N staging		
N0	38	54.3%
N1	17	24.3%
N2a	0	
N2b	9	12.9%
N2c	1	1.4%
Margin		
Free	38	54.3%
Close	31	44.3%
Positive	1	1.4%
Perineural involvement		
None	56	80%
Present	14	20%
Lymphovascular involvement		
None	65	92.9%
Present	5	7.1%
Extracapsular extension		
None	65	92.9%
Present	5	7.1%
Level I pathological involvement		
Yes	11	15.7%
None	59	84.3%
Tumor recurrence		
Locoregional	3	4.3%
Nodal metastasis	2	2.9%
Distant metastasis	8	11.4%

### Vascular supply in clinical practice

**Figure 2:**
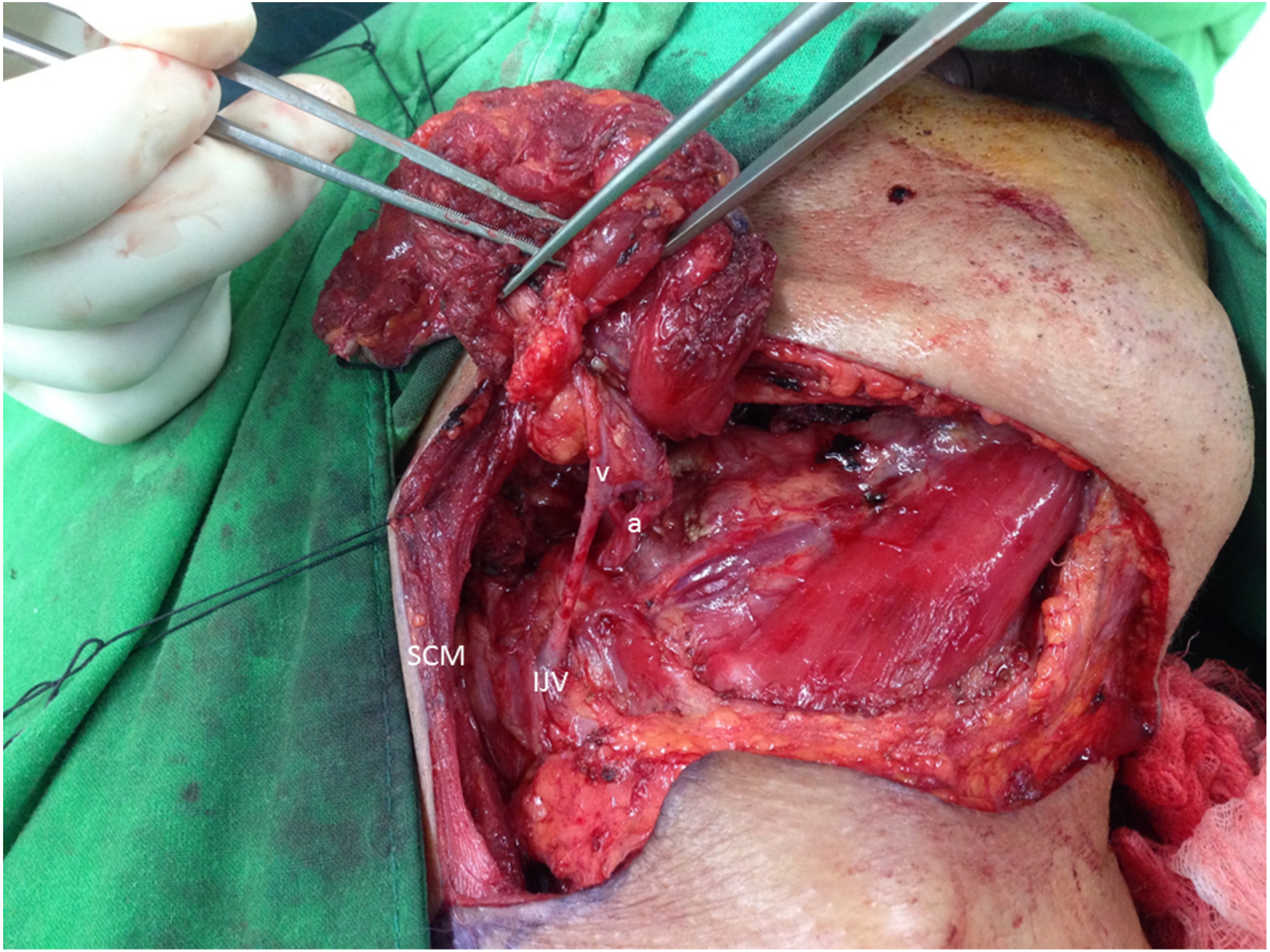
Well-known vasculature of the SF. The submental vein (v) drains into the IJV. The submental artery arises from the facial artery (a). SCM, sternocleidomastoid muscle.

**Figure 3:**
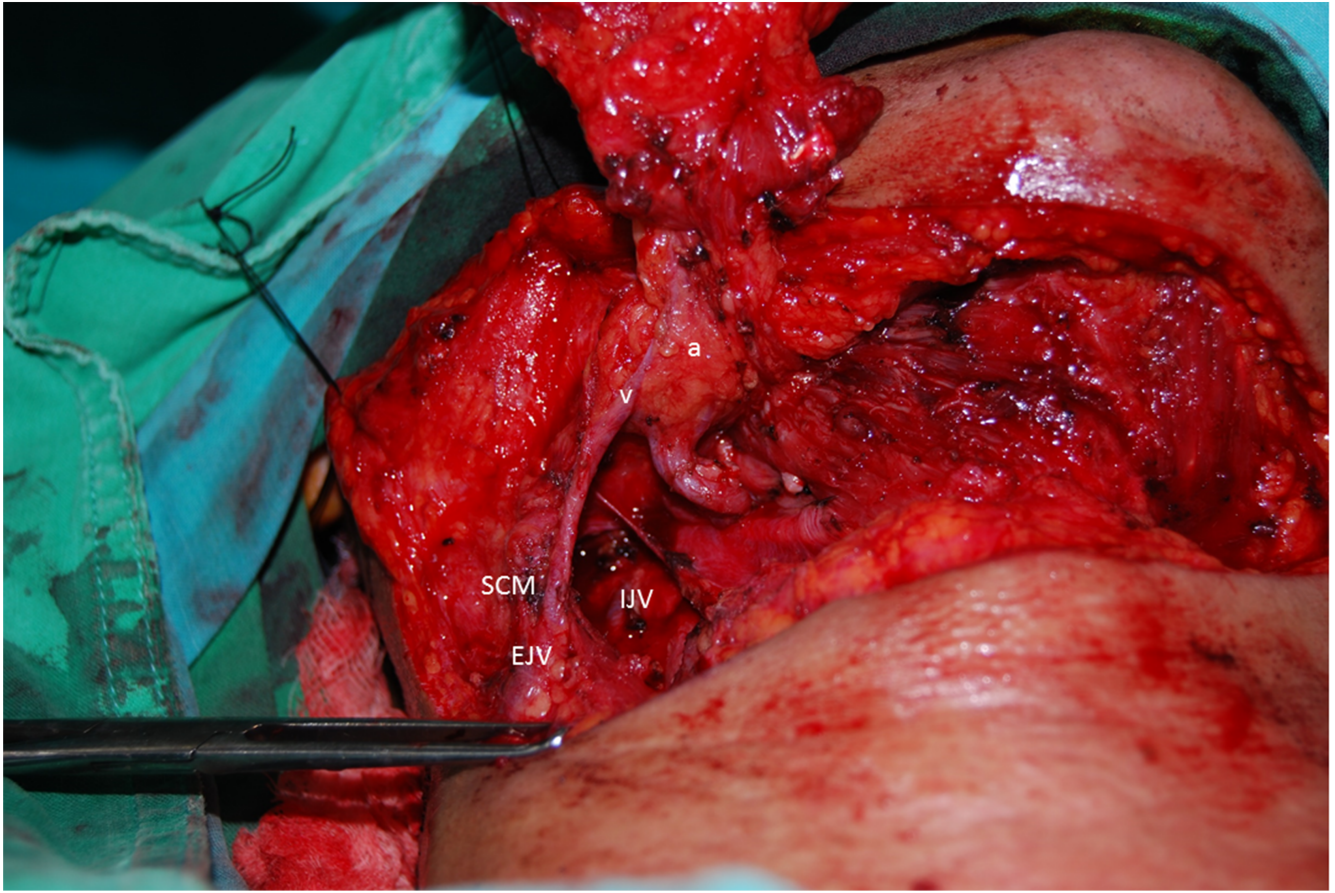
Uncommon pattern of submental venous return. The submental vein (v) drains into the EJV, and the submental artery arises from the facial artery (a). SCM, sternocleidomastoid muscle.

All of the submental arteries in this study originated from the facial artery. However, the submental vein presented a diverse appearance as well as different types of drainage patterns during the operations. Most of our patients’ submental veins originated from the IJV, consistent with the results of well-known studies in the literature (73%) (Fig. 2). However, 19 patients (27%) showed that the submental vein as well as the facial vein both directly drained into the anterior or posterior branch of the EJV (Fig. 3).

Common facial vein drainage into the subcutaneous jugular venous system showed different patterns. According to our 70 live surgeries, drainage to the EJV was classified into three types (I, II and III) based on whether similar anastomosis, shapes and connections occurred between the two veins of the EJV and anterior jugular vein (AJV) (Table 3).

**Figure 4:**
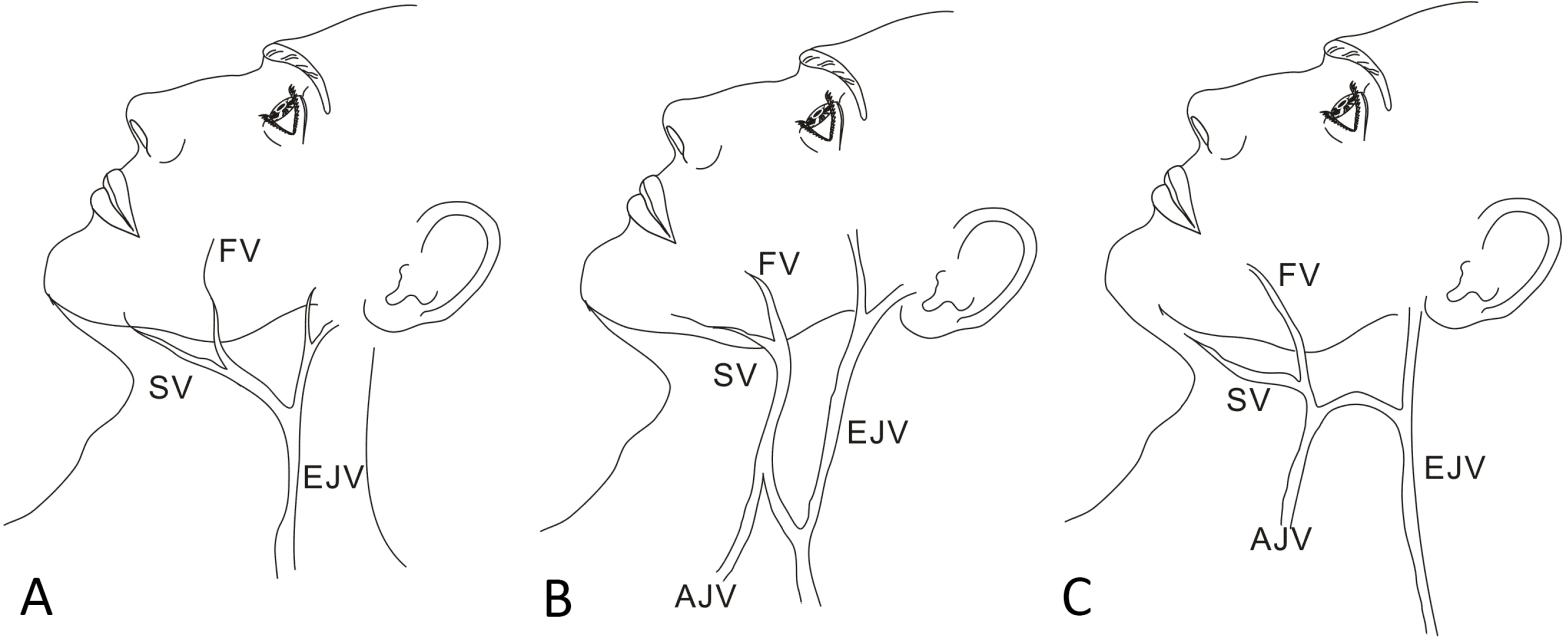
Schematic of venous drainage patterns into the EJV. (A) Type I: V-shaped pattern of the submental venous (SV) return, with the common facial vein (FV) draining directly into the EJV without anastomosis to the AJV. (B) Type II: N-shaped pattern of the submental venous return, with the common facial vein draining anteriorly to the AJV and posteriorly into the anterior branch of the EJV. (C) Type III: H-shaped pattern of the submental venous return, with the common facial vein draining inferiorly to the AJV and horizontally into the EJV.

Figure 4A shows a type I drainage pattern (15 patients), which revealed a V-shaped pattern of submental venous return, with the common facial vein draining directly into the EJV without anastomosis to the AJV. Figure 4B shows a type II drainage pattern (three patients), which revealed an N-shaped pattern of submental venous return, with the common facial vein draining anteriorly into the AJV and posteriorly into the anterior branch of the EJV.

Figure 4C shows a type III drainage pattern (1 patient), which revealed an H-shaped pattern of submental venous return, with the common facial vein draining inferiorly into the AJV and horizontally into the EJV. Both types II and III had anastomosis to the AJV.

## Discussion

This study reported the venous return of the SF based on 70 cases. All of the submental arteries observed originated from the facial artery; however, the submental veins could return to either the IJV (*n* = 51, 72.9%) or the EJV (*n* = 27, 27.1%). The results showed that this uncommon EJV drainage pattern might not represent a simple variation and should be the focus of additional attention. Although this particular drainage pattern has not been widely presented in previous reports, our data suggested that preservation of the IJV and the EJV is mandatory during SF harvesting. The results of this study clearly indicate that the vascular anatomy is a determining factor in successful SF raising. Damage of the potential venous return can occur during neck dissection; thus, SF survival following reconstruction can be affected, which explains the exclusion of several potential participants from our study and underscores the clinical importance of the vascular anatomy in successful SF raising.

**Table 3:** Venous drainage patterns of SFs.

Venous drainage pattern	Number
IJV	51
EJV	
Type I	15
Type II	3
Type III	1

Although the SF can be harvested as a free flap, it is more frequently harvested as a pedicled flap. Therefore, the vasculature of the flap is critical to the survival of the flap. Previous studies have shown that the submental artery branches from the facial artery, which originates from the external carotid artery. However, the submental vein has mainly been described as draining into the IJV (Magden et al., 2004). Two cadaveric studies from India presented variations in drainage to the EJV (approximately 9%) (D’Silva, Pulakunta & Potu, 2008; Gupta et al., 2003). Our data showed that 27% of the study population, which was entirely from Taiwan, presented such a pattern, suggesting that racial/regional factors may affect the drainage pattern of the submental vein.

Variations in the drainage of the common facial vein into the subcutaneous jugular venous system were noted in this study. Most patients with drainage into the EJV were classified with type I drainage patterns with a V shape. During neck dissection, we must carefully preserve the EJV to preserve the venous return of the SF because another anastomosis venous return is not available for the submental vein. If damage to the EJV occurs, the choice of reconstruction might be limited to a free flap or pectoralis major flap, which not only increase the surgical time and anesthesia risk but also result in a new wound. However, the common facial vein drains directly into the EJV without anastomosis to the AJV so that the procedure of tracheostomy does not damage the AJV, which might represent a probable pathway of venous return of the SF. The type II drainage pattern with an N shape and the type III drainage pattern with an H shape both drain into the AJV and EJV in different directions. Compared with type I, the type II and type III present anastomosis to the AJV. If a tracheostomy procedure and neck dissection are required, we must carefully dissect the AJV and EJV to ensure the survival of the flap. Therefore, understanding venous anomalies and variations in the superficial venous return is of great clinical importance. Compared with Gupta’s study from India, we found horizontal connections between two longitudinal veins that formed an H-shaped pattern. However, this H-shaped (type III) pattern was not detected in Indian cadaver dissection, which might provide additional evidence of racial and regional differences in anatomy (Gupta et al., 2003).

Our results indicated that careful preservation of the EJV during surgery is beneficial to patients and important for successful outcomes; therefore, surgeons should be aware of this phenomenon when performing SF raising and neck dissection.

The original surgical technique for SF was proposed by Martin et al. (1993), and a key aspect of the technique includes separating the vascular pedicle from the submandibular gland while preserving the gland. The described procedure required the dissection of small vessels with advanced techniques, and it was riskier for less-experienced surgeons. Thus, Patel, Bayles & Hayden (2007) modified the procedure by including the mylohyoid muscle during SF harvesting. The modified procedure avoided vessel dissection, which decreased the technical difficulty. Furthermore, when the submandibular gland is removed, the vascular pedicle is better viewed during surgery. In the current study, variations in the venous return were observed when the submandibular gland was removed, suggesting that submandibular gland removal is critical for SF harvesting.

SFs have represented a good reconstruction option for head and neck defects since the procedure was first reported. A summary of the clinical applications of SFs in the literature indicates that the recipient sites include the cheek, lower lip, orbit, forehead, auricular, temporal, parotid, mouth floor, alveolus, cervical esophagus, glottis, tongue, preauricular, maxilla, ear canal, mentum, larynx, retromolar trigone, hard palate, infraorbital, jaw, parotid, mastoid, parapharyngeal space and lateral skull base (Magden et al., 2004; Martin et al., 1993; Pinar, Govsa & Bilge, 2005; Pistre et al., 2001; D’Silva, Pulakunta & Potu, 2008; Gupta et al., 2003; Patel, Bayles & Hayden, 2007; Ferrari et al., 2014; Demir, Velidedeoglu & Çelebioglu, 2005; Tan, Atik & Parmaksizoglu, 2007; Abouchadi et al., 2007; Hayden, Nagel & Donald, 2014; Howard et al., 2016; Liu et al., 2013; Taghinia et al., 2009; Tassinari et al., 2010; Vural & Suen, 2000; Lee et al., 2013a; Lee et al., 2013b; Howard et al., 2014; Husso et al., 2015).

### Advantages and disadvantages

The present findings could contribute to a greater understanding of the variations of SF venous return. We conducted a pilot anatomical study with a large number of participants in live surgeries. We also provided regional differences in anatomy in submental vein drainage, which could be of clinical importance in head and neck reconstruction. However, the design of the present study was not without limitations. First, the study was a retrospective case series and might have been subject to selection bias. Second, certain experts believe that the reversed-flow SFs increases the reliability of reconstruction. Kim et al. (2002) proposed the application of reversed-flow SFs in head and neck reconstruction, especially in midface reconstructions. These authors ligated the proximal facial artery and vein at the branching point of the SF so that the blood supply flowed in a reverse-flow pattern supplied by the distal facial pedicle. In addition, the digastric muscle must be included in the flap to prevent venous congestion. Faltaous & Yetman (1996) reported that the submental vessels were located deep in the anterior belly of the digastric muscle in 70% of cadaver dissections; thus, a number of surgeons believe that they should include this muscle as part of the SF to prevent flap failure. In fact, methods that include our SF procedure and reversed-flow SF require a better understanding of the surgical anatomy to ensure more reliable reconstructions, thus indicating that the vascular anatomy is a determining factor for successful SF raising. We are hopeful that future research will provide larger-scale cadaveric anatomic dissection information and a comparison of all previously published case series. Third, another limitation was related to the debate regarding the oncologic safety of SFs. SFs should be more suitable in certain circumstances, such as N0 neck conditions, although this is relatively rare in patients requiring reconstruction after tumor resection. In our study, 54.3% of patients were classified as having an N0 neck condition. When level I lymph node micrometastasis is suspected, several studies have recommended that surgeons should exclude SFs and consider other reconstructive options. Moreover, reports indicated that frozen sections of the suspicious nodes should be carefully examined before flap harvesting (Pistre et al., 2001; Hayden, Nagel & Donald, 2014; Liu et al., 2013). However, Howard et al. (2014) reported five cases of reconstructions with SFs, even when level I micrometastasis was observed; none of the patients showed local recurrence related to occult metastasis with the flap. Oncological safety can also be enhanced by sentinel lymph node biopsy, which allows the neck condition close to the flap pedicle to be examined (Husso et al., 2015). In our case series, one patient was suspected to have regional metastasis, and local recurrence was not observed. Our results indicated that the pedicled SF could be a reliable reconstruction option with appropriate management of level I lymph nodes. The supraclavicular flap could be a perfect alternative to the SF. However, the disadvantage of using the supraclavicular flap is that additional incision is needed. Finally, the application of SFs in soft palate areas is restricted, unlike the radial forearm flap.

## Conclusions

The current study illustrated variations in the submental vein and showed that drainage could occur into the IJV or the EJV. The percentage of patients with variations in EJV drainage was 27%, which was higher than the percentage in previous reports. Therefore, we recommend that preservation of the IJV and EJV should be mandatory to ensure successful SF raising, SF survival and reconstructions. Moreover, the vascular anatomy is a determining factor in SF surgery.

##  Supplemental Information

10.7717/peerj.3606/supp-1 Data S1

10.7717/peerj.3606/supp-2 Supplemental Information 1 ICH6 GCP regulations The committe of TSGH is organized and operates in accordance with ICH6 GCP regulations and guideline.

## Additional Information and Declarations

### Data Availability

The following information was supplied regarding data availability: For ethical reasons to maintain confidentiality, the de-identified raw data has been supplied as a Supplemental File.
