# Supplementary material for: Vascular anatomy is a determining factor of successful submental flap raising: a retrospective study of 70 clinical cases
Source: PeerJ. 2017 Sep 19;5:e3606. doi: 10.7717/peerj.3606 (PMC5609627; doi:10.7717/peerj.3606)
Supplement: Supplemental Information 1 — The committe of TSGH is organized and operates in accordance with ICH6 GCP regulations and guideline. [file peerj-05-3606-s002.pdf]

INTERNATIONAL CONFERENCE ON HARMONISATION OF TECHNICAL REQUIREMENTS FOR REGISTRATION OF PHARMACEUTICALS FOR HUMAN USE

ICH HARMONISED TRIPARTITE GUIDELINE

GUIDELINE FOR GOOD CLINICAL PRACTICE
E6(R1)

Current *Step 4* version

dated 10 June 1996

(including the Post Step 4 corrections)

This Guideline has been developed by the appropriate ICH Expert Working Group and has been subject to consultation by the regulatory parties, in accordance with the ICH Process. At Step 4 of the Process the final draft is recommended for adoption to the regulatory bodies of the European Union, Japan and USA.

E6(R1)
Document History

First Codification	History	Date	New Codification **November 2005**
E6	Approval by the Steering Committee under *Step 2* and release for public consultation.	27 April 1995	E6
E6	Approval by the Steering Committee under *Step 4* and recommended for adoption to the three ICH regulatory bodies.	1 May 1996	E6

Current *Step 4* version

E6	Approval by the Steering Committee of *Post-Step 4* editorial corrections.	10 June 1996	E6(R1)

GUIDELINE FOR GOOD CLINICAL PRACTICE

ICH Harmonised Tripartite Guideline

Having reached *Step 4* of the ICH Process at the ICH Steering Committee meeting on 1 May 1996, this guideline is recommended for adoption to the three regulatory parties to ICH

(This document includes the Post Step 4 corrections agreed by the Steering Committee on 10 June 1996)

TABLE OF CONTENTS

INTRODUCTION 1

1. GLOSSARY 2

2. THE PRINCIPLES OF ICH GCP 8

3. INSTITUTIONAL REVIEW BOARD/INDEPENDENT ETHICS COMMITTEE (IRB/IEC) 9

3.1 Responsibilities 9

3.2 Composition, Functions and Operations 11

3.3 Procedures 11

3.4 Records 12

4. INVESTIGATOR 12

4.1 Investigator's Qualifications and Agreements 12

4.2 Adequate Resources 12

4.3 Medical Care of Trial Subjects 13

4.4 Communication with IRB/IEC 13

4.5 Compliance with Protocol 13

4.6 Investigational Product(s) 14

4.7 Randomization Procedures and Unblinding 15

4.8 Informed Consent of Trial Subjects 15

4.9 Records and Reports 18

4.10 Progress Reports 19

4.11 Safety Reporting 19

4.12 Premature Termination or Suspension of a Trial 19

4.13 Final Report(s) by Investigator 20

5. SPONSOR 20

5.1 Quality Assurance and Quality Control 20

5.2 Contract Research Organization (CRO) 20

5.3 Medical Expertise 21

5.4 Trial Design 21

5.5 Trial Management, Data Handling, and Record Keeping 21

5.6 Investigator Selection ... 22
5.7 Allocation of Responsibilities ... 23
5.8 Compensation to Subjects and Investigators ... 23
5.9 Financing ... 23
5.10 Notification/Submission to Regulatory Authority(ies) ... 23
5.11 Confirmation of Review by IRB/IEC ... 23
5.12 Information on Investigational Product(s) ... 24
5.13 Manufacturing, Packaging, Labelling, and Coding Investigational Product(s) . 24
5.14 Supplying and Handling Investigational Product(s) ... 24
5.15 Record Access ... 25
5.16 Safety Information ... 25
5.17 Adverse Drug Reaction Reporting ... 26
5.18 Monitoring ... 26
5.18.1 Purpose ... 26
5.18.2 Selection and Qualifications of Monitors ... 26
5.18.3 Extent and Nature of Monitoring ... 26
5.18.4 Monitor's Responsibilities ... 26
5.18.5 Monitoring Procedures ... 28
5.18.6 Monitoring Report ... 28
5.19 Audit ... 28
5.19.1 Purpose ... 29
5.19.2 Selection and Qualification of Auditors ... 29
5.19.3 Auditing Procedures ... 29
5.20 Noncompliance ... 29
5.21 Premature Termination or Suspension of a Trial ... 30
5.22 Clinical Trial/Study Reports ... 30
5.23 Multicentre Trials ... 30
6. CLINICAL TRIAL PROTOCOL AND PROTOCOL AMENDMENT(S) ... 30
6.1 General Information ... 30
6.2 Background Information ... 31
6.3 Trial Objectives and Purpose ... 31
6.4 Trial Design ... 31
6.5 Selection and Withdrawal of Subjects ... 32
6.6 Treatment of Subjects ... 32
6.7 Assessment of Efficacy ... 32
6.8 Assessment of Safety ... 32

6.9 Statistics 32
6.10 Direct Access to Source Data/Documents 33
6.11 Quality Control and Quality Assurance 33
6.12 Ethics 33
6.13 Data Handling and Record Keeping 33
6.14 Financing and Insurance 33
6.15 Publication Policy 33
6.16 Supplements 33
7. INVESTIGATOR'S BROCHURE 34
7.1 Introduction 34
7.2 General Considerations 35
7.2.1 Title Page 35
7.2.2 Confidentiality Statement 35
7.3 Contents of the Investigator's Brochure 35
7.3.1 Table of Contents 35
7.3.2 Summary 35
7.3.3 Introduction 35
7.3.4 Physical, Chemical, and Pharmaceutical Properties and Formulation 35
7.3.5 Nonclinical Studies 36
7.3.6 Effects in Humans 37
7.3.7 Summary of Data and Guidance for the Investigator 38
7.4 APPENDIX 1: 39
7.5 APPENDIX 2: 40
8. ESSENTIAL DOCUMENTS FOR THE CONDUCT OF A CLINICAL TRIAL 41
8.1 Introduction 41
8.2 Before the Clinical Phase of the Trial Commences 42
8.3 During the Clinical Conduct of the Trial 46
8.4 After Completion or Termination of the Trial 52

GUIDELINE FOR GOOD CLINICAL PRACTICE

INTRODUCTION

Good Clinical Practice (GCP) is an international ethical and scientific quality standard for designing, conducting, recording and reporting trials that involve the participation of human subjects. Compliance with this standard provides public assurance that the rights, safety and well-being of trial subjects are protected, consistent with the principles that have their origin in the Declaration of Helsinki, and that the clinical trial data are credible.

The objective of this ICH GCP Guideline is to provide a unified standard for the European Union (EU), Japan and the United States to facilitate the mutual acceptance of clinical data by the regulatory authorities in these jurisdictions.

The guideline was developed with consideration of the current good clinical practices of the European Union, Japan, and the United States, as well as those of Australia, Canada, the Nordic countries and the World Health Organization (WHO).

This guideline should be followed when generating clinical trial data that are intended to be submitted to regulatory authorities.

The principles established in this guideline may also be applied to other clinical investigations that may have an impact on the safety and well-being of human subjects.

1. GLOSSARY

1.1 Adverse Drug Reaction (ADR)

In the pre-approval clinical experience with a new medicinal product or its new usages, particularly as the therapeutic dose(s) may not be established: all noxious and unintended responses to a medicinal product related to any dose should be considered adverse drug reactions. The phrase responses to a medicinal product means that a causal relationship between a medicinal product and an adverse event is at least a reasonable possibility, i.e. the relationship cannot be ruled out.

Regarding marketed medicinal products: a response to a drug which is noxious and unintended and which occurs at doses normally used in man for prophylaxis, diagnosis, or therapy of diseases or for modification of physiological function (see the ICH Guideline for Clinical Safety Data Management: Definitions and Standards for Expedited Reporting).

1.2 Adverse Event (AE)

Any untoward medical occurrence in a patient or clinical investigation subject administered a pharmaceutical product and which does not necessarily have a causal relationship with this treatment. An adverse event (AE) can therefore be any unfavourable and unintended sign (including an abnormal laboratory finding), symptom, or disease temporally associated with the use of a medicinal (investigational) product, whether or not related to the medicinal (investigational) product (see the ICH Guideline for Clinical Safety Data Management: Definitions and Standards for Expedited Reporting).

1.3 Amendment (to the protocol)

See Protocol Amendment.

1.4 Applicable Regulatory Requirement(s)

Any law(s) and regulation(s) addressing the conduct of clinical trials of investigational products.

1.5 Approval (in relation to Institutional Review Boards)

The affirmative decision of the IRB that the clinical trial has been reviewed and may be conducted at the institution site within the constraints set forth by the IRB, the institution, Good Clinical Practice (GCP), and the applicable regulatory requirements.

1.6 Audit

A systematic and independent examination of trial related activities and documents to determine whether the evaluated trial related activities were conducted, and the data were recorded, analyzed and accurately reported according to the protocol, sponsor's standard operating procedures (SOPs), Good Clinical Practice (GCP), and the applicable regulatory requirement(s).

1.7 Audit Certificate

A declaration of confirmation by the auditor that an audit has taken place.

1.8 Audit Report

A written evaluation by the sponsor's auditor of the results of the audit.

1.9 Audit Trail

Documentation that allows reconstruction of the course of events.

1.10 Blinding/Masking

A procedure in which one or more parties to the trial are kept unaware of the treatment assignment(s). Single-blinding usually refers to the subject(s) being unaware, and double-blinding usually refers to the subject(s), investigator(s), monitor, and, in some cases, data analyst(s) being unaware of the treatment assignment(s).

1.11 Case Report Form (CRF)

A printed, optical, or electronic document designed to record all of the protocol required information to be reported to the sponsor on each trial subject.

1.12 Clinical Trial/Study

Any investigation in human subjects intended to discover or verify the clinical, pharmacological and/or other pharmacodynamic effects of an investigational product(s), and/or to identify any adverse reactions to an investigational product(s), and/or to study absorption, distribution, metabolism, and excretion of an investigational product(s) with the object of ascertaining its safety and/or efficacy. The terms clinical trial and clinical study are synonymous.

1.13 Clinical Trial/Study Report

A written description of a trial/study of any therapeutic, prophylactic, or diagnostic agent conducted in human subjects, in which the clinical and statistical description, presentations, and analyses are fully integrated into a single report (see the ICH Guideline for Structure and Content of Clinical Study Reports).

1.14 Comparator (Product)

An investigational or marketed product (i.e., active control), or placebo, used as a reference in a clinical trial.

1.15 Compliance (in relation to trials)

Adherence to all the trial-related requirements, Good Clinical Practice (GCP) requirements, and the applicable regulatory requirements.

1.16 Confidentiality

Prevention of disclosure, to other than authorized individuals, of a sponsor's proprietary information or of a subject's identity.

1.17 Contract

A written, dated, and signed agreement between two or more involved parties that sets out any arrangements on delegation and distribution of tasks and obligations and, if appropriate, on financial matters. The protocol may serve as the basis of a contract.

1.18 Coordinating Committee

A committee that a sponsor may organize to coordinate the conduct of a multicentre trial.

1.19 Coordinating Investigator

An investigator assigned the responsibility for the coordination of investigators at different centres participating in a multicentre trial.

1.20 Contract Research Organization (CRO)

A person or an organization (commercial, academic, or other) contracted by the sponsor to perform one or more of a sponsor's trial-related duties and functions.

1.21 Direct Access

Permission to examine, analyze, verify, and reproduce any records and reports that are important to evaluation of a clinical trial. Any party (e.g., domestic and foreign regulatory authorities, sponsor's monitors and auditors) with direct access should take all reasonable precautions within the constraints of the applicable regulatory requirement(s) to maintain the confidentiality of subjects' identities and sponsor’s proprietary information.

1.22 Documentation

All records, in any form (including, but not limited to, written, electronic, magnetic, and optical records, and scans, x-rays, and electrocardiograms) that describe or record the methods, conduct, and/or results of a trial, the factors affecting a trial, and the actions taken.

1.23 Essential Documents

Documents which individually and collectively permit evaluation of the conduct of a study and the quality of the data produced (see 8. Essential Documents for the Conduct of a Clinical Trial).

1.24 Good Clinical Practice (GCP)

A standard for the design, conduct, performance, monitoring, auditing, recording, analyses, and reporting of clinical trials that provides assurance that the data and reported results are credible and accurate, and that the rights, integrity, and confidentiality of trial subjects are protected.

1.25 Independent Data-Monitoring Committee (IDMC) (Data and Safety Monitoring Board, Monitoring Committee, Data Monitoring Committee)

An independent data-monitoring committee that may be established by the sponsor to assess at intervals the progress of a clinical trial, the safety data, and the critical efficacy endpoints, and to recommend to the sponsor whether to continue, modify, or stop a trial.

1.26 Impartial Witness

A person, who is independent of the trial, who cannot be unfairly influenced by people involved with the trial, who attends the informed consent process if the subject or the subject’s legally acceptable representative cannot read, and who reads the informed consent form and any other written information supplied to the subject.

1.27 Independent Ethics Committee (IEC)

An independent body (a review board or a committee, institutional, regional, national, or supranational), constituted of medical professionals and non-medical members, whose responsibility it is to ensure the protection of the rights, safety and well-being of human subjects involved in a trial and to provide public assurance of that protection, by, among other things, reviewing and approving / providing favourable

opinion on, the trial protocol, the suitability of the investigator(s), facilities, and the methods and material to be used in obtaining and documenting informed consent of the trial subjects.

The legal status, composition, function, operations and regulatory requirements pertaining to Independent Ethics Committees may differ among countries, but should allow the Independent Ethics Committee to act in agreement with GCP as described in this guideline.

1.28 Informed Consent

A process by which a subject voluntarily confirms his or her willingness to participate in a particular trial, after having been informed of all aspects of the trial that are relevant to the subject's decision to participate. Informed consent is documented by means of a written, signed and dated informed consent form.

1.29 Inspection

The act by a regulatory authority(ies) of conducting an official review of documents, facilities, records, and any other resources that are deemed by the authority(ies) to be related to the clinical trial and that may be located at the site of the trial, at the sponsor's and/or contract research organization's (CRO's) facilities, or at other establishments deemed appropriate by the regulatory authority(ies).

1.30 Institution (medical)

Any public or private entity or agency or medical or dental facility where clinical trials are conducted.

1.31 Institutional Review Board (IRB)

An independent body constituted of medical, scientific, and non-scientific members, whose responsibility is to ensure the protection of the rights, safety and well-being of human subjects involved in a trial by, among other things, reviewing, approving, and providing continuing review of trial protocol and amendments and of the methods and material to be used in obtaining and documenting informed consent of the trial subjects.

1.32 Interim Clinical Trial/Study Report

A report of intermediate results and their evaluation based on analyses performed during the course of a trial.

1.33 Investigational Product

A pharmaceutical form of an active ingredient or placebo being tested or used as a reference in a clinical trial, including a product with a marketing authorization when used or assembled (formulated or packaged) in a way different from the approved form, or when used for an unapproved indication, or when used to gain further information about an approved use.

1.34 Investigator

A person responsible for the conduct of the clinical trial at a trial site. If a trial is conducted by a team of individuals at a trial site, the investigator is the responsible leader of the team and may be called the principal investigator. See also Subinvestigator.

1.35 Investigator / Institution

An expression meaning "the investigator and/or institution, where required by the applicable regulatory requirements".

1.36 Investigator's Brochure

A compilation of the clinical and nonclinical data on the investigational product(s) which is relevant to the study of the investigational product(s) in human subjects (see 7. Investigator's Brochure).

1.37 Legally Acceptable Representative

An individual or juridical or other body authorized under applicable law to consent, on behalf of a prospective subject, to the subject's participation in the clinical trial.

1.38 Monitoring

The act of overseeing the progress of a clinical trial, and of ensuring that it is conducted, recorded, and reported in accordance with the protocol, Standard Operating Procedures (SOPs), Good Clinical Practice (GCP), and the applicable regulatory requirement(s).

1.39 Monitoring Report

A written report from the monitor to the sponsor after each site visit and/or other trial-related communication according to the sponsor's SOPs.

1.40 Multicentre Trial

A clinical trial conducted according to a single protocol but at more than one site, and therefore, carried out by more than one investigator.

1.41 Nonclinical Study

Biomedical studies not performed on human subjects.

1.42 Opinion (in relation to Independent Ethics Committee)

The judgement and/or the advice provided by an Independent Ethics Committee (IEC).

1.43 Original Medical Record

See Source Documents.

1.44 Protocol

A document that describes the objective(s), design, methodology, statistical considerations, and organization of a trial. The protocol usually also gives the background and rationale for the trial, but these could be provided in other protocol referenced documents. Throughout the ICH GCP Guideline the term protocol refers to protocol and protocol amendments.

1.45 Protocol Amendment

A written description of a change(s) to or formal clarification of a protocol.

1.46 Quality Assurance (QA)

All those planned and systematic actions that are established to ensure that the trial is performed and the data are generated, documented (recorded), and reported in compliance with Good Clinical Practice (GCP) and the applicable regulatory requirement(s).

1.47 Quality Control (QC)

The operational techniques and activities undertaken within the quality assurance system to verify that the requirements for quality of the trial-related activities have been fulfilled.

1.48 Randomization

The process of assigning trial subjects to treatment or control groups using an element of chance to determine the assignments in order to reduce bias.

1.49 Regulatory Authorities

Bodies having the power to regulate. In the ICH GCP guideline the expression Regulatory Authorities includes the authorities that review submitted clinical data and those that conduct inspections (see 1.29). These bodies are sometimes referred to as competent authorities.

1.50 Serious Adverse Event (SAE) or Serious Adverse Drug Reaction (Serious ADR)

Any untoward medical occurrence that at any dose:

- results in death,

- is life-threatening,

- requires inpatient hospitalization or prolongation of existing hospitalization,

- results in persistent or significant disability/incapacity,

or

- is a congenital anomaly/birth defect

(see the ICH Guideline for Clinical Safety Data Management: Definitions and Standards for Expedited Reporting).

1.51 Source Data

All information in original records and certified copies of original records of clinical findings, observations, or other activities in a clinical trial necessary for the reconstruction and evaluation of the trial. Source data are contained in source documents (original records or certified copies).

1.52 Source Documents

Original documents, data, and records (e.g., hospital records, clinical and office charts, laboratory notes, memoranda, subjects' diaries or evaluation checklists, pharmacy dispensing records, recorded data from automated instruments, copies or transcriptions certified after verification as being accurate copies, microfiches, photographic negatives, microfilm or magnetic media, x-rays, subject files, and records kept at the pharmacy, at the laboratories and at medico-technical departments involved in the clinical trial).

1.53 Sponsor

An individual, company, institution, or organization which takes responsibility for the initiation, management, and/or financing of a clinical trial.

1.54 Sponsor-Investigator

An individual who both initiates and conducts, alone or with others, a clinical trial, and under whose immediate direction the investigational product is administered to, dispensed to, or used by a subject. The term does not include any person other than an individual (e.g., it does not include a corporation or an agency). The obligations of a sponsor-investigator include both those of a sponsor and those of an investigator.

1.55 Standard Operating Procedures (SOPs)

Detailed, written instructions to achieve uniformity of the performance of a specific function.

1.56 Subinvestigator

Any individual member of the clinical trial team designated and supervised by the investigator at a trial site to perform critical trial-related procedures and/or to make important trial-related decisions (e.g., associates, residents, research fellows). See also Investigator.

1.57 Subject/Trial Subject

An individual who participates in a clinical trial, either as a recipient of the investigational product(s) or as a control.

1.58 Subject Identification Code

A unique identifier assigned by the investigator to each trial subject to protect the subject's identity and used in lieu of the subject's name when the investigator reports adverse events and/or other trial related data.

1.59 Trial Site

The location(s) where trial-related activities are actually conducted.

1.60 Unexpected Adverse Drug Reaction

An adverse reaction, the nature or severity of which is not consistent with the applicable product information (e.g., Investigator's Brochure for an unapproved investigational product or package insert/summary of product characteristics for an approved product) (see the ICH Guideline for Clinical Safety Data Management: Definitions and Standards for Expedited Reporting).

1.61 Vulnerable Subjects

Individuals whose willingness to volunteer in a clinical trial may be unduly influenced by the expectation, whether justified or not, of benefits associated with participation, or of a retaliatory response from senior members of a hierarchy in case of refusal to participate. Examples are members of a group with a hierarchical structure, such as medical, pharmacy, dental, and nursing students, subordinate hospital and laboratory personnel, employees of the pharmaceutical industry, members of the armed forces, and persons kept in detention. Other vulnerable subjects include patients with incurable diseases, persons in nursing homes, unemployed or impoverished persons, patients in emergency situations, ethnic minority groups, homeless persons, nomads, refugees, minors, and those incapable of giving consent.

1.62 Well-being (of the trial subjects)

The physical and mental integrity of the subjects participating in a clinical trial.

2. THE PRINCIPLES OF ICH GCP

2.1 Clinical trials should be conducted in accordance with the ethical principles that have their origin in the Declaration of Helsinki, and that are consistent with GCP and the applicable regulatory requirement(s).

2.2 Before a trial is initiated, foreseeable risks and inconveniences should be weighed against the anticipated benefit for the individual trial subject and society. A trial should be initiated and continued only if the anticipated benefits justify the risks.

2.3 The rights, safety, and well-being of the trial subjects are the most important considerations and should prevail over interests of science and society.

2.4 The available nonclinical and clinical information on an investigational product should be adequate to support the proposed clinical trial.

2.5 Clinical trials should be scientifically sound, and described in a clear, detailed protocol.

2.6 A trial should be conducted in compliance with the protocol that has received prior institutional review board (IRB)/independent ethics committee (IEC) approval/favourable opinion.

2.7 The medical care given to, and medical decisions made on behalf of, subjects should always be the responsibility of a qualified physician or, when appropriate, of a qualified dentist.

2.8 Each individual involved in conducting a trial should be qualified by education, training, and experience to perform his or her respective task(s).

2.9 Freely given informed consent should be obtained from every subject prior to clinical trial participation.

2.10 All clinical trial information should be recorded, handled, and stored in a way that allows its accurate reporting, interpretation and verification.

2.11 The confidentiality of records that could identify subjects should be protected, respecting the privacy and confidentiality rules in accordance with the applicable regulatory requirement(s).

2.12 Investigational products should be manufactured, handled, and stored in accordance with applicable good manufacturing practice (GMP). They should be used in accordance with the approved protocol.

2.13 Systems with procedures that assure the quality of every aspect of the trial should be implemented.

3. INSTITUTIONAL REVIEW BOARD/INDEPENDENT ETHICS COMMITTEE (IRB/IEC)

3.1 Responsibilities

3.1.1 An IRB/IEC should safeguard the rights, safety, and well-being of all trial subjects. Special attention should be paid to trials that may include vulnerable subjects.

3.1.2 The IRB/IEC should obtain the following documents:

trial protocol(s)/amendment(s), written informed consent form(s) and consent form updates that the investigator proposes for use in the trial, subject recruitment procedures (e.g. advertisements), written information to be provided to subjects, Investigator's Brochure (IB), available safety information, information about payments and compensation available to subjects, the investigator's current curriculum vitae and/or other documentation evidencing qualifications, and any other documents that the IRB/IEC may need to fulfil its responsibilities.

The IRB/IEC should review a proposed clinical trial within a reasonable time and document its views in writing, clearly identifying the trial, the documents reviewed and the dates for the following:

- approval/favourable opinion;

- modifications required prior to its approval/favourable opinion;

- disapproval / negative opinion; and

- termination/suspension of any prior approval/favourable opinion.

3.1.3 The IRB/IEC should consider the qualifications of the investigator for the proposed trial, as documented by a current curriculum vitae and/or by any other relevant documentation the IRB/IEC requests.

3.1.4 The IRB/IEC should conduct continuing review of each ongoing trial at intervals appropriate to the degree of risk to human subjects, but at least once per year.

3.1.5 The IRB/IEC may request more information than is outlined in paragraph 4.8.10 be given to subjects when, in the judgement of the IRB/IEC, the additional information would add meaningfully to the protection of the rights, safety and/or well-being of the subjects.

3.1.6 When a non-therapeutic trial is to be carried out with the consent of the subject's legally acceptable representative (see 4.8.12, 4.8.14), the IRB/IEC should determine that the proposed protocol and/or other document(s) adequately addresses relevant ethical concerns and meets applicable regulatory requirements for such trials.

3.1.7 Where the protocol indicates that prior consent of the trial subject or the subject's legally acceptable representative is not possible (see 4.8.15), the IRB/IEC should determine that the proposed protocol and/or other document(s) adequately addresses relevant ethical concerns and meets applicable regulatory requirements for such trials (i.e. in emergency situations).

3.1.8 The IRB/IEC should review both the amount and method of payment to subjects to assure that neither presents problems of coercion or undue influence on the trial subjects. Payments to a subject should be prorated and not wholly contingent on completion of the trial by the subject.

3.1.9 The IRB/IEC should ensure that information regarding payment to subjects, including the methods, amounts, and schedule of payment to trial subjects, is set forth in the written informed consent form and any other written information to be provided to subjects. The way payment will be prorated should be specified.

3.2 Composition, Functions and Operations

3.2.1 The IRB/IEC should consist of a reasonable number of members, who collectively have the qualifications and experience to review and evaluate the science, medical aspects, and ethics of the proposed trial. It is recommended that the IRB/IEC should include:

(a) At least five members.

(b) At least one member whose primary area of interest is in a nonscientific area.

(c) At least one member who is independent of the institution/trial site.

Only those IRB/IEC members who are independent of the investigator and the sponsor of the trial should vote/provide opinion on a trial-related matter.

A list of IRB/IEC members and their qualifications should be maintained.

3.2.2 The IRB/IEC should perform its functions according to written operating procedures, should maintain written records of its activities and minutes of its meetings, and should comply with GCP and with the applicable regulatory requirement(s).

3.2.3 An IRB/IEC should make its decisions at announced meetings at which at least a quorum, as stipulated in its written operating procedures, is present.

3.2.4 Only members who participate in the IRB/IEC review and discussion should vote/provide their opinion and/or advise.

3.2.5 The investigator may provide information on any aspect of the trial, but should not participate in the deliberations of the IRB/IEC or in the vote/opinion of the IRB/IEC.

3.2.6 An IRB/IEC may invite nonmembers with expertise in special areas for assistance.

3.3 Procedures

The IRB/IEC should establish, document in writing, and follow its procedures, which should include:

3.3.1 Determining its composition (names and qualifications of the members) and the authority under which it is established.

3.3.2 Scheduling, notifying its members of, and conducting its meetings.

3.3.3 Conducting initial and continuing review of trials.

3.3.4 Determining the frequency of continuing review, as appropriate.

3.3.5 Providing, according to the applicable regulatory requirements, expedited review and approval/favourable opinion of minor change(s) in ongoing trials that have the approval/favourable opinion of the IRB/IEC.

3.3.6 Specifying that no subject should be admitted to a trial before the IRB/IEC issues its written approval/favourable opinion of the trial.

3.3.7 Specifying that no deviations from, or changes of, the protocol should be initiated without prior written IRB/IEC approval/favourable opinion of an appropriate amendment, except when necessary to eliminate immediate hazards to the subjects or when the change(s) involves only logistical or

administrative aspects of the trial (e.g., change of monitor(s), telephone number(s)) (see 4.5.2).

3.3.8 Specifying that the investigator should promptly report to the IRB/IEC:

(a) Deviations from, or changes of, the protocol to eliminate immediate hazards to the trial subjects (see 3.3.7, 4.5.2, 4.5.4).

(b) Changes increasing the risk to subjects and/or affecting significantly the conduct of the trial (see 4.10.2).

(c) All adverse drug reactions (ADRs) that are both serious and unexpected.

(d) New information that may affect adversely the safety of the subjects or the conduct of the trial.

3.3.9 Ensuring that the IRB/IEC promptly notify in writing the investigator/institution concerning:

(a) Its trial-related decisions/opinions.

(b) The reasons for its decisions/opinions.

(c) Procedures for appeal of its decisions/opinions.

3.4 Records

The IRB/IEC should retain all relevant records (e.g., written procedures, membership lists, lists of occupations/affiliations of members, submitted documents, minutes of meetings, and correspondence) for a period of at least 3 years after completion of the trial and make them available upon request from the regulatory authority(ies).

The IRB/IEC may be asked by investigators, sponsors or regulatory authorities to provide its written procedures and membership lists.

4. INVESTIGATOR

4.1 Investigator's Qualifications and Agreements

4.1.1 The investigator(s) should be qualified by education, training, and experience to assume responsibility for the proper conduct of the trial, should meet all the qualifications specified by the applicable regulatory requirement(s), and should provide evidence of such qualifications through up-to-date curriculum vitae and/or other relevant documentation requested by the sponsor, the IRB/IEC, and/or the regulatory authority(ies).

4.1.2 The investigator should be thoroughly familiar with the appropriate use of the investigational product(s), as described in the protocol, in the current Investigator's Brochure, in the product information and in other information sources provided by the sponsor.

4.1.3 The investigator should be aware of, and should comply with, GCP and the applicable regulatory requirements.

4.1.4 The investigator/institution should permit monitoring and auditing by the sponsor, and inspection by the appropriate regulatory authority(ies).

4.1.5 The investigator should maintain a list of appropriately qualified persons to whom the investigator has delegated significant trial-related duties.

4.2 Adequate Resources

4.2.1 The investigator should be able to demonstrate (e.g., based on retrospective data) a potential for recruiting the required number of suitable subjects within the agreed recruitment period.

4.2.2 The investigator should have sufficient time to properly conduct and complete the trial within the agreed trial period.

4.2.3 The investigator should have available an adequate number of qualified staff and adequate facilities for the foreseen duration of the trial to conduct the trial properly and safely.

4.2.4 The investigator should ensure that all persons assisting with the trial are adequately informed about the protocol, the investigational product(s), and their trial-related duties and functions.

4.3 Medical Care of Trial Subjects

4.3.1 A qualified physician (or dentist, when appropriate), who is an investigator or a sub-investigator for the trial, should be responsible for all trial-related medical (or dental) decisions.

4.3.2 During and following a subject's participation in a trial, the investigator/institution should ensure that adequate medical care is provided to a subject for any adverse events, including clinically significant laboratory values, related to the trial. The investigator/institution should inform a subject when medical care is needed for intercurrent illness(es) of which the investigator becomes aware.

4.3.3 It is recommended that the investigator inform the subject's primary physician about the subject's participation in the trial if the subject has a primary physician and if the subject agrees to the primary physician being informed.

4.3.4 Although a subject is not obliged to give his/her reason(s) for withdrawing prematurely from a trial, the investigator should make a reasonable effort to ascertain the reason(s), while fully respecting the subject's rights.

4.4 Communication with IRB/IEC

4.4.1 Before initiating a trial, the investigator/institution should have written and dated approval/favourable opinion from the IRB/IEC for the trial protocol, written informed consent form, consent form updates, subject recruitment procedures (e.g., advertisements), and any other written information to be provided to subjects.

4.4.2 As part of the investigator's/institution’s written application to the IRB/IEC, the investigator/institution should provide the IRB/IEC with a current copy of the Investigator's Brochure. If the Investigator's Brochure is updated during the trial, the investigator/institution should supply a copy of the updated Investigator’s Brochure to the IRB/IEC.

4.4.3 During the trial the investigator/institution should provide to the IRB/IEC all documents subject to review.

4.5 Compliance with Protocol

4.5.1 The investigator/institution should conduct the trial in compliance with the protocol agreed to by the sponsor and, if required, by the regulatory authority(ies) and which was given approval/favourable opinion by the

IRB/IEC. The investigator/institution and the sponsor should sign the protocol, or an alternative contract, to confirm agreement.

4.5.2 The investigator should not implement any deviation from, or changes of the protocol without agreement by the sponsor and prior review and documented approval/favourable opinion from the IRB/IEC of an amendment, except where necessary to eliminate an immediate hazard(s) to trial subjects, or when the change(s) involves only logistical or administrative aspects of the trial (e.g., change in monitor(s), change of telephone number(s)).

4.5.3 The investigator, or person designated by the investigator, should document and explain any deviation from the approved protocol.

4.5.4 The investigator may implement a deviation from, or a change of, the protocol to eliminate an immediate hazard(s) to trial subjects without prior IRB/IEC approval/favourable opinion. As soon as possible, the implemented deviation or change, the reasons for it, and, if appropriate, the proposed protocol amendment(s) should be submitted:

(a) to the IRB/IEC for review and approval/favourable opinion,

(b) to the sponsor for agreement and, if required,

(c) to the regulatory authority(ies).

4.6 Investigational Product(s)

4.6.1 Responsibility for investigational product(s) accountability at the trial site(s) rests with the investigator/institution.

4.6.2 Where allowed/required, the investigator/institution may/should assign some or all of the investigator's/institution's duties for investigational product(s) accountability at the trial site(s) to an appropriate pharmacist or another appropriate individual who is under the supervision of the investigator/institution..

4.6.3 The investigator/institution and/or a pharmacist or other appropriate individual, who is designated by the investigator/institution, should maintain records of the product's delivery to the trial site, the inventory at the site, the use by each subject, and the return to the sponsor or alternative disposition of unused product(s). These records should include dates, quantities, batch/serial numbers, expiration dates (if applicable), and the unique code numbers assigned to the investigational product(s) and trial subjects. Investigators should maintain records that document adequately that the subjects were provided the doses specified by the protocol and reconcile all investigational product(s) received from the sponsor.

4.6.4 The investigational product(s) should be stored as specified by the sponsor (see 5.13.2 and 5.14.3) and in accordance with applicable regulatory requirement(s).

4.6.5 The investigator should ensure that the investigational product(s) are used only in accordance with the approved protocol.

4.6.6 The investigator, or a person designated by the investigator/institution, should explain the correct use of the investigational product(s) to each subject and should check, at intervals appropriate for the trial, that each subject is following the instructions properly.

4.7 Randomization Procedures and Unblinding

The investigator should follow the trial's randomization procedures, if any, and should ensure that the code is broken only in accordance with the protocol. If the trial is blinded, the investigator should promptly document and explain to the sponsor any premature unblinding (e.g., accidental unblinding, unblinding due to a serious adverse event) of the investigational product(s).

4.8 Informed Consent of Trial Subjects

4.8.1 In obtaining and documenting informed consent, the investigator should comply with the applicable regulatory requirement(s), and should adhere to GCP and to the ethical principles that have their origin in the Declaration of Helsinki. Prior to the beginning of the trial, the investigator should have the IRB/IEC's written approval/favourable opinion of the written informed consent form and any other written information to be provided to subjects.

4.8.2 The written informed consent form and any other written information to be provided to subjects should be revised whenever important new information becomes available that may be relevant to the subject’s consent. Any revised written informed consent form, and written information should receive the IRB/IEC's approval/favourable opinion in advance of use. The subject or the subject’s legally acceptable representative should be informed in a timely manner if new information becomes available that may be relevant to the subject’s willingness to continue participation in the trial. The communication of this information should be documented.

4.8.3 Neither the investigator, nor the trial staff, should coerce or unduly influence a subject to participate or to continue to participate in a trial.

4.8.4 None of the oral and written information concerning the trial, including the written informed consent form, should contain any language that causes the subject or the subject's legally acceptable representative to waive or to appear to waive any legal rights, or that releases or appears to release the investigator, the institution, the sponsor, or their agents from liability for negligence.

4.8.5 The investigator, or a person designated by the investigator, should fully inform the subject or, if the subject is unable to provide informed consent, the subject's legally acceptable representative, of all pertinent aspects of the trial including the written information and the approval/ favourable opinion by the IRB/IEC.

4.8.6 The language used in the oral and written information about the trial, including the written informed consent form, should be as non-technical as practical and should be understandable to the subject or the subject's legally acceptable representative and the impartial witness, where applicable.

4.8.7 Before informed consent may be obtained, the investigator, or a person designated by the investigator, should provide the subject or the subject's legally acceptable representative ample time and opportunity to inquire about details of the trial and to decide whether or not to participate in the trial. All questions about the trial should be answered to the satisfaction of the subject or the subject's legally acceptable representative.

4.8.8 Prior to a subject’s participation in the trial, the written informed consent form should be signed and personally dated by the subject or by the subject's legally

acceptable representative, and by the person who conducted the informed consent discussion.

4.8.9 If a subject is unable to read or if a legally acceptable representative is unable to read, an impartial witness should be present during the entire informed consent discussion. After the written informed consent form and any other written information to be provided to subjects, is read and explained to the subject or the subject's legally acceptable representative, and after the subject or the subject's legally acceptable representative has orally consented to the subject's participation in the trial and, if capable of doing so, has signed and personally dated the informed consent form, the witness should sign and personally date the consent form. By signing the consent form, the witness attests that the information in the consent form and any other written information was accurately explained to, and apparently understood by, the subject or the subject's legally acceptable representative, and that informed consent was freely given by the subject or the subject's legally acceptable representative.

4.8.10 Both the informed consent discussion and the written informed consent form and any other written information to be provided to subjects should include explanations of the following:

(a) That the trial involves research.

(b) The purpose of the trial.

(c) The trial treatment(s) and the probability for random assignment to each treatment.

(d) The trial procedures to be followed, including all invasive procedures.

(e) The subject's responsibilities.

(f) Those aspects of the trial that are experimental.

(g) The reasonably foreseeable risks or inconveniences to the subject and, when applicable, to an embryo, fetus, or nursing infant.

(h) The reasonably expected benefits. When there is no intended clinical benefit to the subject, the subject should be made aware of this.

(i) The alternative procedure(s) or course(s) of treatment that may be available to the subject, and their important potential benefits and risks.

(j) The compensation and/or treatment available to the subject in the event of trial-related injury.

(k) The anticipated prorated payment, if any, to the subject for participating in the trial.

(l) The anticipated expenses, if any, to the subject for participating in the trial.

(m) That the subject's participation in the trial is voluntary and that the subject may refuse to participate or withdraw from the trial, at any time, without penalty or loss of benefits to which the subject is otherwise entitled.

(n) That the monitor(s), the auditor(s), the IRB/IEC, and the regulatory authority(ies) will be granted direct access to the subject's original medical records for verification of clinical trial procedures and/or data, without violating the confidentiality of the subject, to the extent permitted by the

applicable laws and regulations and that, by signing a written informed consent form, the subject or the subject's legally acceptable representative is authorizing such access.

(o) That records identifying the subject will be kept confidential and, to the extent permitted by the applicable laws and/or regulations, will not be made publicly available. If the results of the trial are published, the subject's identity will remain confidential.

(p) That the subject or the subject's legally acceptable representative will be informed in a timely manner if information becomes available that may be relevant to the subject's willingness to continue participation in the trial.

(q) The person(s) to contact for further information regarding the trial and the rights of trial subjects, and whom to contact in the event of trial-related injury.

(r) The foreseeable circumstances and/or reasons under which the subject's participation in the trial may be terminated.

(s) The expected duration of the subject's participation in the trial.

(t) The approximate number of subjects involved in the trial.

4.8.11 Prior to participation in the trial, the subject or the subject's legally acceptable representative should receive a copy of the signed and dated written informed consent form and any other written information provided to the subjects. During a subject's participation in the trial, the subject or the subject's legally acceptable representative should receive a copy of the signed and dated consent form updates and a copy of any amendments to the written information provided to subjects.

4.8.12 When a clinical trial (therapeutic or non-therapeutic) includes subjects who can only be enrolled in the trial with the consent of the subject's legally acceptable representative (e.g., minors, or patients with severe dementia), the subject should be informed about the trial to the extent compatible with the subject's understanding and, if capable, the subject should sign and personally date the written informed consent.

4.8.13 Except as described in 4.8.14, a non-therapeutic trial (i.e. a trial in which there is no anticipated direct clinical benefit to the subject), should be conducted in subjects who personally give consent and who sign and date the written informed consent form.

4.8.14 Non-therapeutic trials may be conducted in subjects with consent of a legally acceptable representative provided the following conditions are fulfilled:

(a) The objectives of the trial can not be met by means of a trial in subjects who can give informed consent personally.

(b) The foreseeable risks to the subjects are low.

(c) The negative impact on the subject's well-being is minimized and low.

(d) The trial is not prohibited by law.

(e) The approval/favourable opinion of the IRB/IEC is expressly sought on the inclusion of such subjects, and the written approval/ favourable opinion covers this aspect.

Such trials, unless an exception is justified, should be conducted in patients having a disease or condition for which the investigational product is intended. Subjects in these trials should be particularly closely monitored and should be withdrawn if they appear to be unduly distressed.

4.8.15 In emergency situations, when prior consent of the subject is not possible, the consent of the subject's legally acceptable representative, if present, should be requested. When prior consent of the subject is not possible, and the subject's legally acceptable representative is not available, enrolment of the subject should require measures described in the protocol and/or elsewhere, with documented approval/favourable opinion by the IRB/IEC, to protect the rights, safety and well-being of the subject and to ensure compliance with applicable regulatory requirements. The subject or the subject's legally acceptable representative should be informed about the trial as soon as possible and consent to continue and other consent as appropriate (see 4.8.10) should be requested.

4.9 Records and Reports

4.9.1 The investigator should ensure the accuracy, completeness, legibility, and timeliness of the data reported to the sponsor in the CRFs and in all required reports.

4.9.2 Data reported on the CRF, that are derived from source documents, should be consistent with the source documents or the discrepancies should be explained.

4.9.3 Any change or correction to a CRF should be dated, initialed, and explained (if necessary) and should not obscure the original entry (i.e. an audit trail should be maintained); this applies to both written and electronic changes or corrections (see 5.18.4 (n)). Sponsors should provide guidance to investigators and/or the investigators' designated representatives on making such corrections. Sponsors should have written procedures to assure that changes or corrections in CRFs made by sponsor's designated representatives are documented, are necessary, and are endorsed by the investigator. The investigator should retain records of the changes and corrections.

4.9.4 The investigator/institution should maintain the trial documents as specified in Essential Documents for the Conduct of a Clinical Trial (see 8.) and as required by the applicable regulatory requirement(s). The investigator/institution should take measures to prevent accidental or premature destruction of these documents.

4.9.5 Essential documents should be retained until at least 2 years after the last approval of a marketing application in an ICH region and until there are no pending or contemplated marketing applications in an ICH region or at least 2 years have elapsed since the formal discontinuation of clinical development of the investigational product. These documents should be retained for a longer period however if required by the applicable regulatory requirements or by an agreement with the sponsor. It is the responsibility of the sponsor to inform the investigator/institution as to when these documents no longer need to be retained (see 5.5.12).

4.9.6 The financial aspects of the trial should be documented in an agreement between the sponsor and the investigator/institution.

4.9.7 Upon request of the monitor, auditor, IRB/IEC, or regulatory authority, the investigator/institution should make available for direct access all requested trial-related records.

4.10 Progress Reports

4.10.1 The investigator should submit written summaries of the trial status to the IRB/IEC annually, or more frequently, if requested by the IRB/IEC.

4.10.2 The investigator should promptly provide written reports to the sponsor, the IRB/IEC (see 3.3.8) and, where applicable, the institution on any changes significantly affecting the conduct of the trial, and/or increasing the risk to subjects.

4.11 Safety Reporting

4.11.1 All serious adverse events (SAEs) should be reported immediately to the sponsor except for those SAEs that the protocol or other document (e.g., Investigator's Brochure) identifies as not needing immediate reporting. The immediate reports should be followed promptly by detailed, written reports. The immediate and follow-up reports should identify subjects by unique code numbers assigned to the trial subjects rather than by the subjects' names, personal identification numbers, and/or addresses. The investigator should also comply with the applicable regulatory requirement(s) related to the reporting of unexpected serious adverse drug reactions to the regulatory authority(ies) and the IRB/IEC.

4.11.2 Adverse events and/or laboratory abnormalities identified in the protocol as critical to safety evaluations should be reported to the sponsor according to the reporting requirements and within the time periods specified by the sponsor in the protocol.

4.11.3 For reported deaths, the investigator should supply the sponsor and the IRB/IEC with any additional requested information (e.g., autopsy reports and terminal medical reports).

4.12 Premature Termination or Suspension of a Trial

If the trial is prematurely terminated or suspended for any reason, the investigator/institution should promptly inform the trial subjects, should assure appropriate therapy and follow-up for the subjects, and, where required by the applicable regulatory requirement(s), should inform the regulatory authority(ies). In addition:

4.12.1 If the investigator terminates or suspends a trial without prior agreement of the sponsor, the investigator should inform the institution where applicable, and the investigator/institution should promptly inform the sponsor and the IRB/IEC, and should provide the sponsor and the IRB/IEC a detailed written explanation of the termination or suspension.

4.12.2 If the sponsor terminates or suspends a trial (see 5.21), the investigator should promptly inform the institution where applicable and the investigator/institution should promptly inform the IRB/IEC and provide the IRB/IEC a detailed written explanation of the termination or suspension.

4.12.3 If the IRB/IEC terminates or suspends its approval/favourable opinion of a trial (see 3.1.2 and 3.3.9), the investigator should inform the institution where applicable and the investigator/institution should promptly notify the sponsor and provide the sponsor with a detailed written explanation of the termination or suspension.

4.13 Final Report(s) by Investigator

Upon completion of the trial, the investigator, where applicable, should inform the institution; the investigator/institution should provide the IRB/IEC with a summary of the trial's outcome, and the regulatory authority(ies) with any reports required.

5. SPONSOR

5.1 Quality Assurance and Quality Control

5.1.1 The sponsor is responsible for implementing and maintaining quality assurance and quality control systems with written SOPs to ensure that trials are conducted and data are generated, documented (recorded), and reported in compliance with the protocol, GCP, and the applicable regulatory requirement(s).

5.1.2 The sponsor is responsible for securing agreement from all involved parties to ensure direct access (see 1.21) to all trial related sites, source data/documents , , and reports for the purpose of monitoring and auditing by the sponsor, and inspection by domestic and foreign regulatory authorities.

5.1.3 Quality control should be applied to each stage of data handling to ensure that all data are reliable and have been processed correctly.

5.1.4 Agreements, made by the sponsor with the investigator/institution and any other parties involved with the clinical trial, should be in writing, as part of the protocol or in a separate agreement.

5.2 Contract Research Organization (CRO)

5.2.1 A sponsor may transfer any or all of the sponsor's trial-related duties and functions to a CRO, but the ultimate responsibility for the quality and integrity of the trial data always resides with the sponsor. The CRO should implement quality assurance and quality control.

5.2.2 Any trial-related duty and function that is transferred to and assumed by a CRO should be specified in writing.

5.2.3 Any trial-related duties and functions not specifically transferred to and assumed by a CRO are retained by the sponsor.

5.2.4 All references to a sponsor in this guideline also apply to a CRO to the extent that a CRO has assumed the trial related duties and functions of a sponsor.

5.3 Medical Expertise

The sponsor should designate appropriately qualified medical personnel who will be readily available to advise on trial related medical questions or problems. If necessary, outside consultant(s) may be appointed for this purpose.

5.4 Trial Design

5.4.1 The sponsor should utilize qualified individuals (e.g. biostatisticians, clinical pharmacologists, and physicians) as appropriate, throughout all stages of the trial process, from designing the protocol and CRFs and planning the analyses to analyzing and preparing interim and final clinical trial reports.

5.4.2 For further guidance: Clinical Trial Protocol and Protocol Amendment(s) (see 6.), the ICH Guideline for Structure and Content of Clinical Study Reports, and other appropriate ICH guidance on trial design, protocol and conduct.

5.5 Trial Management, Data Handling, and Record Keeping

5.5.1 The sponsor should utilize appropriately qualified individuals to supervise the overall conduct of the trial, to handle the data, to verify the data, to conduct the statistical analyses, and to prepare the trial reports.

5.5.2 The sponsor may consider establishing an independent data-monitoring committee (IDMC) to assess the progress of a clinical trial, including the safety data and the critical efficacy endpoints at intervals, and to recommend to the sponsor whether to continue, modify, or stop a trial. The IDMC should have written operating procedures and maintain written records of all its meetings.

5.5.3 When using electronic trial data handling and/or remote electronic trial data systems, the sponsor should:

(a) Ensure and document that the electronic data processing system(s) conforms to the sponsor's established requirements for completeness, accuracy, reliability, and consistent intended performance (i.e. validation).

(b) Maintains SOPs for using these systems.

(c) Ensure that the systems are designed to permit data changes in such a way that the data changes are documented and that there is no deletion of entered data (i.e. maintain an audit trail, data trail, edit trail).

(d) Maintain a security system that prevents unauthorized access to the data.

(e) Maintain a list of the individuals who are authorized to make data changes (see 4.1.5 and 4.9.3).

(f) Maintain adequate backup of the data.

(g) Safeguard the blinding, if any (e.g. maintain the blinding during data entry and processing).

5.5.4 If data are transformed during processing, it should always be possible to compare the original data and observations with the processed data.

5.5.5 The sponsor should use an unambiguous subject identification code (see 1.58) that allows identification of all the data reported for each subject.

5.5.6 The sponsor, or other owners of the data, should retain all of the sponsor-specific essential documents pertaining to the trial (see 8. Essential Documents for the Conduct of a Clinical Trial).

5.5.7 The sponsor should retain all sponsor-specific essential documents in conformance with the applicable regulatory requirement(s) of the country(ies) where the product is approved, and/or where the sponsor intends to apply for approval(s).

5.5.8 If the sponsor discontinues the clinical development of an investigational product (i.e. for any or all indications, routes of administration, or dosage forms), the sponsor should maintain all sponsor-specific essential documents for at least 2 years after formal discontinuation or in conformance with the applicable regulatory requirement(s).

5.5.9 If the sponsor discontinues the clinical development of an investigational product, the sponsor should notify all the trial investigators/institutions and all the regulatory authorities.

5.5.10 Any transfer of ownership of the data should be reported to the appropriate authority(ies), as required by the applicable regulatory requirement(s).

5.5.11 The sponsor specific essential documents should be retained until at least 2 years after the last approval of a marketing application in an ICH region and until there are no pending or contemplated marketing applications in an ICH region or at least 2 years have elapsed since the formal discontinuation of clinical development of the investigational product. These documents should be retained for a longer period however if required by the applicable regulatory requirement(s) or if needed by the sponsor.

5.5.12 The sponsor should inform the investigator(s)/institution(s) in writing of the need for record retention and should notify the investigator(s)/institution(s) in writing when the trial related records are no longer needed.

5.6 Investigator Selection

5.6.1 The sponsor is responsible for selecting the investigator(s)/institution(s). Each investigator should be qualified by training and experience and should have adequate resources (see 4.1, 4.2) to properly conduct the trial for which the investigator is selected. If organization of a coordinating committee and/or selection of coordinating investigator(s) are to be utilized in multicentre trials, their organization and/or selection are the sponsor's responsibility.

5.6.2 Before entering an agreement with an investigator/institution to conduct a trial, the sponsor should provide the investigator(s)/institution(s) with the protocol and an up-to-date Investigator's Brochure, and should provide sufficient time for the investigator/institution to review the protocol and the information provided.

5.6.3 The sponsor should obtain the investigator's/institution's agreement:

(a) to conduct the trial in compliance with GCP, with the applicable regulatory requirement(s) (see 4.1.3), and with the protocol agreed to by the sponsor and given approval/favourable opinion by the IRB/IEC (see 4.5.1);

(b) to comply with procedures for data recording/reporting;

(c) to permit monitoring, auditing and inspection (see 4.1.4) and

(d) to retain the trial related essential documents until the sponsor informs the investigator/institution these documents are no longer needed (see 4.9.4 and 5.5.12).

The sponsor and the investigator/institution should sign the protocol, or an alternative document, to confirm this agreement.

5.7 Allocation of Responsibilities

Prior to initiating a trial, the sponsor should define, establish, and allocate all trial-related duties and functions.

5.8 Compensation to Subjects and Investigators

5.8.1 If required by the applicable regulatory requirement(s), the sponsor should provide insurance or should indemnify (legal and financial coverage) the investigator/the institution against claims arising from the trial, except for claims that arise from malpractice and/or negligence.

5.8.2 The sponsor's policies and procedures should address the costs of treatment of trial subjects in the event of trial-related injuries in accordance with the applicable regulatory requirement(s).

5.8.3 When trial subjects receive compensation, the method and manner of compensation should comply with applicable regulatory requirement(s).

5.9 Financing

The financial aspects of the trial should be documented in an agreement between the sponsor and the investigator/institution.

5.10 Notification/Submission to Regulatory Authority(ies)

Before initiating the clinical trial(s), the sponsor (or the sponsor and the investigator, if required by the applicable regulatory requirement(s)) should submit any required application(s) to the appropriate authority(ies) for review, acceptance, and/or permission (as required by the applicable regulatory requirement(s)) to begin the trial(s). Any notification/submission should be dated and contain sufficient information to identify the protocol.

5.11 Confirmation of Review by IRB/IEC

5.11.1 The sponsor should obtain from the investigator/institution:

(a) The name and address of the investigator's/institution’s IRB/IEC.

(b) A statement obtained from the IRB/IEC that it is organized and operates according to GCP and the applicable laws and regulations.

(c) Documented IRB/IEC approval/favourable opinion and, if requested by the sponsor, a current copy of protocol, written informed consent form(s) and any other written information to be provided to subjects, subject recruiting procedures, and documents related to payments and compensation available to the subjects, and any other documents that the IRB/IEC may have requested.

5.11.2 If the IRB/IEC conditions its approval/favourable opinion upon change(s) in any aspect of the trial, such as modification(s) of the protocol, written informed consent form and any other written information to be provided to subjects, and/or other procedures, the sponsor should obtain from the investigator/institution a copy of the modification(s) made and the date approval/favourable opinion was given by the IRB/IEC.

5.11.3 The sponsor should obtain from the investigator/institution documentation and dates of any IRB/IEC reapprovals/re-evaluations with favourable opinion, and of any withdrawals or suspensions of approval/favourable opinion.

5.12 Information on Investigational Product(s)

5.12.1 When planning trials, the sponsor should ensure that sufficient safety and efficacy data from nonclinical studies and/or clinical trials are available to support human exposure by the route, at the dosages, for the duration, and in the trial population to be studied.

5.12.2 The sponsor should update the Investigator's Brochure as significant new information becomes available (see 7. Investigator's Brochure).

5.13 Manufacturing, Packaging, Labelling, and Coding Investigational Product(s)

5.13.1 The sponsor should ensure that the investigational product(s) (including active comparator(s) and placebo, if applicable) is characterized as appropriate to the stage of development of the product(s), is manufactured in accordance with any applicable GMP, and is coded and labelled in a manner that protects the blinding, if applicable. In addition, the labelling should comply with applicable regulatory requirement(s).

5.13.2 The sponsor should determine, for the investigational product(s), acceptable storage temperatures, storage conditions (e.g. protection from light), storage times, reconstitution fluids and procedures, and devices for product infusion, if any. The sponsor should inform all involved parties (e.g. monitors, investigators, pharmacists, storage managers) of these determinations.

5.13.3 The investigational product(s) should be packaged to prevent contamination and unacceptable deterioration during transport and storage.

5.13.4 In blinded trials, the coding system for the investigational product(s) should include a mechanism that permits rapid identification of the product(s) in case of a medical emergency, but does not permit undetectable breaks of the blinding.

5.13.5 If significant formulation changes are made in the investigational or comparator product(s) during the course of clinical development, the results of any additional studies of the formulated product(s) (e.g. stability, dissolution rate, bioavailability) needed to assess whether these changes would significantly alter the pharmacokinetic profile of the product should be available prior to the use of the new formulation in clinical trials.

5.14 Supplying and Handling Investigational Product(s)

5.14.1 The sponsor is responsible for supplying the investigator(s)/institution(s) with the investigational product(s).

5.14.2 The sponsor should not supply an investigator/institution with the investigational product(s) until the sponsor obtains all required documentation (e.g. approval/favourable opinion from IRB/IEC and regulatory authority(ies)).

5.14.3 The sponsor should ensure that written procedures include instructions that the investigator/institution should follow for the handling and storage of investigational product(s) for the trial and documentation thereof. The procedures should address adequate and safe receipt, handling, storage, dispensing, retrieval of unused product from subjects, and return of unused investigational product(s) to the sponsor (or alternative disposition if authorized by the sponsor and in compliance with the applicable regulatory requirement(s)).

5.14.4 The sponsor should:

(a) Ensure timely delivery of investigational product(s) to the investigator(s).

(b) Maintain records that document shipment, receipt, disposition, return, and destruction of the investigational product(s) (see 8. Essential Documents for the Conduct of a Clinical Trial).

(c) Maintain a system for retrieving investigational products and documenting this retrieval (e.g. for deficient product recall, reclaim after trial completion, expired product reclaim).

(d) Maintain a system for the disposition of unused investigational product(s) and for the documentation of this disposition.

5.14.5 The sponsor should:

(a) Take steps to ensure that the investigational product(s) are stable over the period of use.

(b) Maintain sufficient quantities of the investigational product(s) used in the trials to reconfirm specifications, should this become necessary, and maintain records of batch sample analyses and characteristics. To the extent stability permits, samples should be retained either until the analyses of the trial data are complete or as required by the applicable regulatory requirement(s), whichever represents the longer retention period.

5.15 Record Access

5.15.1 The sponsor should ensure that it is specified in the protocol or other written agreement that the investigator(s)/institution(s) provide direct access to source data/documents for trial-related monitoring, audits, IRB/IEC review, and regulatory inspection.

5.15.2 The sponsor should verify that each subject has consented, in writing, to direct access to his/her original medical records for trial-related monitoring, audit, IRB/IEC review, and regulatory inspection.

5.16 Safety Information

5.16.1 The sponsor is responsible for the ongoing safety evaluation of the investigational product(s).

5.16.2 The sponsor should promptly notify all concerned investigator(s)/institution(s) and the regulatory authority(ies) of findings that could affect adversely the

safety of subjects, impact the conduct of the trial, or alter the IRB/IEC's approval/favourable opinion to continue the trial.

5.17 Adverse Drug Reaction Reporting

5.17.1 The sponsor should expedite the reporting to all concerned investigator(s)/institutions(s), to the IRB(s)/IEC(s), where required, and to the regulatory authority(ies) of all adverse drug reactions (ADRs) that are both serious and unexpected.

5.17.2 Such expedited reports should comply with the applicable regulatory requirement(s) and with the ICH Guideline for Clinical Safety Data Management: Definitions and Standards for Expedited Reporting.

5.17.3 The sponsor should submit to the regulatory authority(ies) all safety updates and periodic reports, as required by applicable regulatory requirement(s).

5.18 Monitoring

5.18.1 Purpose

The purposes of trial monitoring are to verify that:

(a) The rights and well-being of human subjects are protected.

(b) The reported trial data are accurate, complete, and verifiable from source documents.

(c) The conduct of the trial is in compliance with the currently approved protocol/amendment(s), with GCP, and with the applicable regulatory requirement(s).

5.18.2 Selection and Qualifications of Monitors

(a) Monitors should be appointed by the sponsor.

(b) Monitors should be appropriately trained, and should have the scientific and/or clinical knowledge needed to monitor the trial adequately. A monitor’s qualifications should be documented.

(c) Monitors should be thoroughly familiar with the investigational product(s), the protocol, written informed consent form and any other written information to be provided to subjects, the sponsor’s SOPs, GCP, and the applicable regulatory requirement(s).

5.18.3 Extent and Nature of Monitoring

The sponsor should ensure that the trials are adequately monitored. The sponsor should determine the appropriate extent and nature of monitoring. The determination of the extent and nature of monitoring should be based on considerations such as the objective, purpose, design, complexity, blinding, size, and endpoints of the trial. In general there is a need for on-site monitoring, before, during, and after the trial; however in exceptional circumstances the sponsor may determine that central monitoring in conjunction with procedures such as investigators’ training and meetings, and extensive written guidance can assure appropriate conduct of the trial in accordance with GCP. Statistically controlled sampling may be an acceptable method for selecting the data to be verified.

5.18.4 Monitor's Responsibilities

The monitor(s) in accordance with the sponsor's requirements should ensure that the trial is conducted and documented properly by carrying out the following activities when relevant and necessary to the trial and the trial site:

(a) Acting as the main line of communication between the sponsor and the investigator.

(b) Verifying that the investigator has adequate qualifications and resources (see 4.1, 4.2, 5.6) and remain adequate throughout the trial period, that facilities, including laboratories, equipment, and staff, are adequate to safely and properly conduct the trial and remain adequate throughout the trial period.

(c) Verifying, for the investigational product(s):

  (i) That storage times and conditions are acceptable, and that supplies are sufficient throughout the trial.

  (ii) That the investigational product(s) are supplied only to subjects who are eligible to receive it and at the protocol specified dose(s).

  (iii) That subjects are provided with necessary instruction on properly using, handling, storing, and returning the investigational product(s).

  (iv) That the receipt, use, and return of the investigational product(s) at the trial sites are controlled and documented adequately.

  (v) That the disposition of unused investigational product(s) at the trial sites complies with applicable regulatory requirement(s) and is in accordance with the sponsor.

(d) Verifying that the investigator follows the approved protocol and all approved amendment(s), if any.

(e) Verifying that written informed consent was obtained before each subject's participation in the trial.

(f) Ensuring that the investigator receives the current Investigator's Brochure, all documents, and all trial supplies needed to conduct the trial properly and to comply with the applicable regulatory requirement(s).

(g) Ensuring that the investigator and the investigator's trial staff are adequately informed about the trial.

(h) Verifying that the investigator and the investigator's trial staff are performing the specified trial functions, in accordance with the protocol and any other written agreement between the sponsor and the investigator/institution, and have not delegated these functions to unauthorized individuals.

(i) Verifying that the investigator is enroling only eligible subjects.

(j) Reporting the subject recruitment rate.

(k) Verifying that source documents and other trial records are accurate, complete, kept up-to-date and maintained.

(l) Verifying that the investigator provides all the required reports, notifications, applications, and submissions, and that these documents are accurate, complete, timely, legible, dated, and identify the trial.

(m) Checking the accuracy and completeness of the CRF entries, source documents and other trial-related records against each other. The monitor specifically should verify that:

(i) The data required by the protocol are reported accurately on the CRFs and are consistent with the source documents.

(ii) Any dose and/or therapy modifications are well documented for each of the trial subjects.

(iii) Adverse events, concomitant medications and intercurrent illnesses are reported in accordance with the protocol on the CRFs.

(iv) Visits that the subjects fail to make, tests that are not conducted, and examinations that are not performed are clearly reported as such on the CRFs.

(v) All withdrawals and dropouts of enrolled subjects from the trial are reported and explained on the CRFs.

(n) Informing the investigator of any CRF entry error, omission, or illegibility. The monitor should ensure that appropriate corrections, additions, or deletions are made, dated, explained (if necessary), and initialled by the investigator or by a member of the investigator's trial staff who is authorized to initial CRF changes for the investigator. This authorization should be documented.

(o) Determining whether all adverse events (AEs) are appropriately reported within the time periods required by GCP, the protocol, the IRB/IEC, the sponsor, and the applicable regulatory requirement(s).

(p) Determining whether the investigator is maintaining the essential documents (see 8. Essential Documents for the Conduct of a Clinical Trial).

(q) Communicating deviations from the protocol, SOPs, GCP, and the applicable regulatory requirements to the investigator and taking appropriate action designed to prevent recurrence of the detected deviations.

5.18.5 Monitoring Procedures

The monitor(s) should follow the sponsor’s established written SOPs as well as those procedures that are specified by the sponsor for monitoring a specific trial.

5.18.6 Monitoring Report

(a) The monitor should submit a written report to the sponsor after each trial-site visit or trial-related communication.

(b) Reports should include the date, site, name of the monitor, and name of the investigator or other individual(s) contacted.

(c) Reports should include a summary of what the monitor reviewed and the monitor's statements concerning the significant findings/facts, deviations and deficiencies, conclusions, actions taken or to be taken and/or actions recommended to secure compliance.

(d) The review and follow-up of the monitoring report with the sponsor should be documented by the sponsor’s designated representative.

5.19 Audit

If or when sponsors perform audits, as part of implementing quality assurance, they should consider:

5.19.1 Purpose

The purpose of a sponsor's audit, which is independent of and separate from routine monitoring or quality control functions, should be to evaluate trial conduct and compliance with the protocol, SOPs, GCP, and the applicable regulatory requirements.

5.19.2 Selection and Qualification of Auditors

(a) The sponsor should appoint individuals, who are independent of the clinical trials/systems, to conduct audits.

(b) The sponsor should ensure that the auditors are qualified by training and experience to conduct audits properly. An auditor's qualifications should be documented.

5.19.3 Auditing Procedures

(a) The sponsor should ensure that the auditing of clinical trials/systems is conducted in accordance with the sponsor's written procedures on what to audit, how to audit, the frequency of audits, and the form and content of audit reports.

(b) The sponsor's audit plan and procedures for a trial audit should be guided by the importance of the trial to submissions to regulatory authorities, the number of subjects in the trial, the type and complexity of the trial, the level of risks to the trial subjects, and any identified problem(s).

(c) The observations and findings of the auditor(s) should be documented.

(d) To preserve the independence and value of the audit function, the regulatory authority(ies) should not routinely request the audit reports. Regulatory authority(ies) may seek access to an audit report on a case by case basis when evidence of serious GCP non-compliance exists, or in the course of legal proceedings.

(e) When required by applicable law or regulation, the sponsor should provide an audit certificate.

5.20 Noncompliance

5.20.1 Noncompliance with the protocol, SOPs, GCP, and/or applicable regulatory requirement(s) by an investigator/institution, or by member(s) of the sponsor's staff should lead to prompt action by the sponsor to secure compliance.

5.20.2 If the monitoring and/or auditing identifies serious and/or persistent noncompliance on the part of an investigator/institution, the sponsor should terminate the investigator's/institution's participation in the trial. When an investigator's/institution's participation is terminated because of noncompliance, the sponsor should notify promptly the regulatory authority(ies).

5.21 Premature Termination or Suspension of a Trial

If a trial is prematurely terminated or suspended, the sponsor should promptly inform the investigators/institutions, and the regulatory authority(ies) of the termination or suspension and the reason(s) for the termination or suspension. The IRB/IEC should also be informed promptly and provided the reason(s) for the termination or suspension by the sponsor or by the investigator/institution, as specified by the applicable regulatory requirement(s).

5.22 Clinical Trial/Study Reports

Whether the trial is completed or prematurely terminated, the sponsor should ensure that the clinical trial reports are prepared and provided to the regulatory agency(ies) as required by the applicable regulatory requirement(s). The sponsor should also ensure that the clinical trial reports in marketing applications meet the standards of the ICH Guideline for Structure and Content of Clinical Study Reports. (NOTE: The ICH Guideline for Structure and Content of Clinical Study Reports specifies that abbreviated study reports may be acceptable in certain cases.)

5.23 Multicentre Trials

For multicentre trials, the sponsor should ensure that:

5.23.1 All investigators conduct the trial in strict compliance with the protocol agreed to by the sponsor and, if required, by the regulatory authority(ies), and given approval/favourable opinion by the IRB/IEC.

5.23.2 The CRFs are designed to capture the required data at all multicentre trial sites. For those investigators who are collecting additional data, supplemental CRFs should also be provided that are designed to capture the additional data.

5.23.3 The responsibilities of coordinating investigator(s) and the other participating investigators are documented prior to the start of the trial.

5.23.4 All investigators are given instructions on following the protocol, on complying with a uniform set of standards for the assessment of clinical and laboratory findings, and on completing the CRFs.

5.23.5 Communication between investigators is facilitated.

6. CLINICAL TRIAL PROTOCOL AND PROTOCOL AMENDMENT(S)

The contents of a trial protocol should generally include the following topics. However, site specific information may be provided on separate protocol page(s), or addressed in a separate agreement, and some of the information listed below may be contained in other protocol referenced documents, such as an Investigator's Brochure.

6.1 General Information

6.1.1 Protocol title, protocol identifying number, and date. Any amendment(s) should also bear the amendment number(s) and date(s).

6.1.2 Name and address of the sponsor and monitor (if other than the sponsor).

6.1.3 Name and title of the person(s) authorized to sign the protocol and the protocol amendment(s) for the sponsor.

6.1.4 Name, title, address, and telephone number(s) of the sponsor's medical expert (or dentist when appropriate) for the trial.

6.1.5 Name and title of the investigator(s) who is (are) responsible for conducting the trial, and the address and telephone number(s) of the trial site(s).

6.1.6 Name, title, address, and telephone number(s) of the qualified physician (or dentist, if applicable), who is responsible for all trial-site related medical (or dental) decisions (if other than investigator).

6.1.7 Name(s) and address(es) of the clinical laboratory(ies) and other medical and/or technical department(s) and/or institutions involved in the trial.

6.2 Background Information

6.2.1 Name and description of the investigational product(s).

6.2.2 A summary of findings from nonclinical studies that potentially have clinical significance and from clinical trials that are relevant to the trial.

6.2.3 Summary of the known and potential risks and benefits, if any, to human subjects.

6.2.4 Description of and justification for the route of administration, dosage, dosage regimen, and treatment period(s).

6.2.5 A statement that the trial will be conducted in compliance with the protocol, GCP and the applicable regulatory requirement(s).

6.2.6 Description of the population to be studied.

6.2.7 References to literature and data that are relevant to the trial, and that provide background for the trial.

6.3 Trial Objectives and Purpose

A detailed description of the objectives and the purpose of the trial.

6.4 Trial Design

The scientific integrity of the trial and the credibility of the data from the trial depend substantially on the trial design. A description of the trial design, should include:

6.4.1 A specific statement of the primary endpoints and the secondary endpoints, if any, to be measured during the trial.

6.4.2 A description of the type/design of trial to be conducted (e.g. double-blind, placebo-controlled, parallel design) and a schematic diagram of trial design, procedures and stages.

6.4.3 A description of the measures taken to minimize/avoid bias, including:

(a) Randomization.

(b) Blinding.

6.4.4 A description of the trial treatment(s) and the dosage and dosage regimen of the investigational product(s). Also include a description of the dosage form, packaging, and labelling of the investigational product(s).

6.4.5 The expected duration of subject participation, and a description of the sequence and duration of all trial periods, including follow-up, if any.

6.4.6 A description of the "stopping rules" or "discontinuation criteria" for individual subjects, parts of trial and entire trial.

6.4.7 Accountability procedures for the investigational product(s), including the placebo(s) and comparator(s), if any.

6.4.8 Maintenance of trial treatment randomization codes and procedures for breaking codes.

6.4.9 The identification of any data to be recorded directly on the CRFs (i.e. no prior written or electronic record of data), and to be considered to be source data.

6.5 Selection and Withdrawal of Subjects

6.5.1 Subject inclusion criteria.

6.5.2 Subject exclusion criteria.

6.5.3 Subject withdrawal criteria (i.e. terminating investigational product treatment/trial treatment) and procedures specifying:

(a) When and how to withdraw subjects from the trial/ investigational product treatment.

(b) The type and timing of the data to be collected for withdrawn subjects.

(c) Whether and how subjects are to be replaced.

(d) The follow-up for subjects withdrawn from investigational product treatment/trial treatment.

6.6 Treatment of Subjects

6.6.1 The treatment(s) to be administered, including the name(s) of all the product(s), the dose(s), the dosing schedule(s), the route/mode(s) of administration, and the treatment period(s), including the follow-up period(s) for subjects for each investigational product treatment/trial treatment group/arm of the trial.

6.6.2 Medication(s)/treatment(s) permitted (including rescue medication) and not permitted before and/or during the trial.

6.6.3 Procedures for monitoring subject compliance.

6.7 Assessment of Efficacy

6.7.1 Specification of the efficacy parameters.

6.7.2 Methods and timing for assessing, recording, and analysing of efficacy parameters.

6.8 Assessment of Safety

6.8.1 Specification of safety parameters.

6.8.2 The methods and timing for assessing, recording, and analysing safety parameters.

6.8.3 Procedures for eliciting reports of and for recording and reporting adverse event and intercurrent illnesses.

6.8.4 The type and duration of the follow-up of subjects after adverse events.

6.9 Statistics

6.9.1 A description of the statistical methods to be employed, including timing of any planned interim analysis(ses).

6.9.2 The number of subjects planned to be enrolled. In multicentre trials, the numbers of enrolled subjects projected for each trial site should be specified. Reason for choice of sample size, including reflections on (or calculations of) the power of the trial and clinical justification.

6.9.3 The level of significance to be used.

6.9.4 Criteria for the termination of the trial.

6.9.5 Procedure for accounting for missing, unused, and spurious data.

6.9.6 Procedures for reporting any deviation(s) from the original statistical plan (any deviation(s) from the original statistical plan should be described and justified in protocol and/or in the final report, as appropriate).

6.9.7 The selection of subjects to be included in the analyses (e.g. all randomized subjects, all dosed subjects, all eligible subjects, evaluable subjects).

6.10 Direct Access to Source Data/Documents

The sponsor should ensure that it is specified in the protocol or other written agreement that the investigator(s)/institution(s) will permit trial-related monitoring, audits, IRB/IEC review, and regulatory inspection(s), providing direct access to source data/documents.

6.11 Quality Control and Quality Assurance

6.12 Ethics

Description of ethical considerations relating to the trial.

6.13 Data Handling and Record Keeping

6.14 Financing and Insurance

Financing and insurance if not addressed in a separate agreement.

6.15 Publication Policy

Publication policy, if not addressed in a separate agreement.

6.16 Supplements

(NOTE: Since the protocol and the clinical trial/study report are closely related, further relevant information can be found in the ICH Guideline for Structure and Content of Clinical Study Reports.)

7. INVESTIGATOR'S BROCHURE

7.1 Introduction

The Investigator's Brochure (IB) is a compilation of the clinical and nonclinical data on the investigational product(s) that are relevant to the study of the product(s) in human subjects. Its purpose is to provide the investigators and others involved in the trial with the information to facilitate their understanding of the rationale for, and their compliance with, many key features of the protocol, such as the dose, dose frequency/interval, methods of administration: and safety monitoring procedures. The IB also provides insight to support the clinical management of the study subjects during the course of the clinical trial. The information should be presented in a concise, simple, objective, balanced, and non-promotional form that enables a clinician, or potential investigator, to understand it and make his/her own unbiased risk-benefit assessment of the appropriateness of the proposed trial. For this reason, a medically qualified person should generally participate in the editing of an IB, but the contents of the IB should be approved by the disciplines that generated the described data.

This guideline delineates the minimum information that should be included in an IB and provides suggestions for its layout. It is expected that the type and extent of information available will vary with the stage of development of the investigational product. If the investigational product is marketed and its pharmacology is widely understood by medical practitioners, an extensive IB may not be necessary. Where permitted by regulatory authorities, a basic product information brochure, package leaflet, or labelling may be an appropriate alternative, provided that it includes current, comprehensive, and detailed information on all aspects of the investigational product that might be of importance to the investigator. If a marketed product is being studied for a new use (i.e., a new indication), an IB specific to that new use should be prepared. The IB should be reviewed at least annually and revised as necessary in compliance with a sponsor's written procedures. More frequent revision may be appropriate depending on the stage of development and the generation of relevant new information. However, in accordance with Good Clinical Practice, relevant new information may be so important that it should be communicated to the investigators, and possibly to the Institutional Review Boards (IRBs)/Independent Ethics Committees (IECs) and/or regulatory authorities before it is included in a revised IB.

Generally, the sponsor is responsible for ensuring that an up-to-date IB is made available to the investigator(s) and the investigators are responsible for providing the up-to-date IB to the responsible IRBs/IECs. In the case of an investigator sponsored trial, the sponsor-investigator should determine whether a brochure is available from the commercial manufacturer. If the investigational product is provided by the sponsor-investigator, then he or she should provide the necessary information to the trial personnel. In cases where preparation of a formal IB is impractical, the sponsor-investigator should provide, as a substitute, an expanded background information section in the trial protocol that contains the minimum current information described in this guideline.

7.2 General Considerations

The IB should include:

7.2.1 Title Page

This should provide the sponsor's name, the identity of each investigational product (i.e., research number, chemical or approved generic name, and trade name(s) where legally permissible and desired by the sponsor), and the release date. It is also suggested that an edition number, and a reference to the number and date of the edition it supersedes, be provided. An example is given in Appendix 1.

7.2.2 Confidentiality Statement

The sponsor may wish to include a statement instructing the investigator/recipients to treat the IB as a confidential document for the sole information and use of the investigator's team and the IRB/IEC.

7.3 Contents of the Investigator's Brochure

The IB should contain the following sections, each with literature references where appropriate:

7.3.1 Table of Contents

An example of the Table of Contents is given in Appendix 2

7.3.2 Summary

A brief summary (preferably not exceeding two pages) should be given, highlighting the significant physical, chemical, pharmaceutical, pharmacological, toxicological, pharmacokinetic, metabolic, and clinical information available that is relevant to the stage of clinical development of the investigational product.

7.3.3 Introduction

A brief introductory statement should be provided that contains the chemical name (and generic and trade name(s) when approved) of the investigational product(s), all active ingredients, the investigational product (s) pharmacological class and its expected position within this class (e.g. advantages), the rationale for performing research with the investigational product(s), and the anticipated prophylactic, therapeutic, or diagnostic indication(s). Finally, the introductory statement should provide the general approach to be followed in evaluating the investigational product.

7.3.4 Physical, Chemical, and Pharmaceutical Properties and Formulation

A description should be provided of the investigational product substance(s) (including the chemical and/or structural formula(e)), and a brief summary should be given of the relevant physical, chemical, and pharmaceutical properties.

To permit appropriate safety measures to be taken in the course of the trial, a description of the formulation(s) to be used, including excipients, should be provided and justified if clinically relevant. Instructions for the storage and handling of the dosage form(s) should also be given.

Any structural similarities to other known compounds should be mentioned.

7.3.5 Nonclinical Studies

Introduction:

The results of all relevant nonclinical pharmacology, toxicology, pharmacokinetic, and investigational product metabolism studies should be provided in summary form. This summary should address the methodology used, the results, and a discussion of the relevance of the findings to the investigated therapeutic and the possible unfavourable and unintended effects in humans.

The information provided may include the following, as appropriate, if known/available:

- Species tested
- Number and sex of animals in each group
- Unit dose (e.g., milligram/kilogram (mg/kg))
- Dose interval
- Route of administration
- Duration of dosing
- Information on systemic distribution
- Duration of post-exposure follow-up
- Results, including the following aspects:
  - Nature and frequency of pharmacological or toxic effects
  - Severity or intensity of pharmacological or toxic effects
  - Time to onset of effects
  - Reversibility of effects
  - Duration of effects
  - Dose response

Tabular format/listings should be used whenever possible to enhance the clarity of the presentation.

The following sections should discuss the most important findings from the studies, including the dose response of observed effects, the relevance to humans, and any aspects to be studied in humans. If applicable, the effective and nontoxic dose findings in the same animal species should be compared (i.e., the therapeutic index should be discussed). The relevance of this information to the proposed human dosing should be addressed. Whenever possible, comparisons should be made in terms of blood/tissue levels rather than on a mg/kg basis.

(a) Nonclinical Pharmacology

A summary of the pharmacological aspects of the investigational product and, where appropriate, its significant metabolites studied in animals, should be included. Such a summary should incorporate studies that assess potential therapeutic activity (e.g. efficacy models, receptor binding, and specificity) as well as those that assess safety (e.g., special studies to assess pharmacological actions other than the intended therapeutic effect(s)).

(b) Pharmacokinetics and Product Metabolism in Animals

A summary of the pharmacokinetics and biological transformation and disposition of the investigational product in all species studied should be given. The discussion of the findings should address the absorption and the local and systemic bioavailability of the investigational product and its metabolites, and their relationship to the pharmacological and toxicological findings in animal species.

(c) Toxicology

A summary of the toxicological effects found in relevant studies conducted in different animal species should be described under the following headings where appropriate:

- Single dose
- Repeated dose
- Carcinogenicity
- Special studies (e.g. irritancy and sensitisation)
- Reproductive toxicity
- Genotoxicity (mutagenicity)

7.3.6 Effects in Humans

Introduction:

A thorough discussion of the known effects of the investigational product(s) in humans should be provided, including information on pharmacokinetics, metabolism, pharmacodynamics, dose response, safety, efficacy, and other pharmacological activities. Where possible, a summary of each completed clinical trial should be provided. Information should also be provided regarding results of any use of the investigational product(s) other than from in clinical trials, such as from experience during marketing.

(a) Pharmacokinetics and Product Metabolism in Humans

- A summary of information on the pharmacokinetics of the investigational product(s) should be presented, including the following, if available:
- Pharmacokinetics (including metabolism, as appropriate, and absorption, plasma protein binding, distribution, and elimination).
- Bioavailability of the investigational product (absolute, where possible, and/or relative) using a reference dosage form.
- Population subgroups (e.g., gender, age, and impaired organ function).
- Interactions (e.g., product-product interactions and effects of food).
- Other pharmacokinetic data (e.g., results of population studies performed within clinical trial(s).

(b) Safety and Efficacy

A summary of information should be provided about the investigational product's/products' (including metabolites, where appropriate) safety, pharmacodynamics, efficacy, and dose response that were obtained from preceding trials in humans (healthy volunteers and/or patients). The implications of this information should be discussed. In cases where a number of clinical trials have been completed, the use of summaries of safety and efficacy across multiple trials by indications in subgroups may provide a clear presentation of the data. Tabular summaries of adverse drug reactions for all the clinical trials (including those for all the studied indications) would be useful. Important differences in adverse drug

reaction patterns/incidences across indications or subgroups should be discussed.

The IB should provide a description of the possible risks and adverse drug reactions to be anticipated on the basis of prior experiences with the product under investigation and with related products. A description should also be provided of the precautions or special monitoring to be done as part of the investigational use of the product(s).

(c) Marketing Experience

The IB should identify countries where the investigational product has been marketed or approved. Any significant information arising from the marketed use should be summarised (e.g., formulations, dosages, routes of administration, and adverse product reactions). The IB should also identify all the countries where the investigational product did not receive approval/registration for marketing or was withdrawn from marketing/registration.

7.3.7 Summary of Data and Guidance for the Investigator

This section should provide an overall discussion of the nonclinical and clinical data, and should summarise the information from various sources on different aspects of the investigational product(s), wherever possible. In this way, the investigator can be provided with the most informative interpretation of the available data and with an assessment of the implications of the information for future clinical trials.

Where appropriate, the published reports on related products should be discussed. This could help the investigator to anticipate adverse drug reactions or other problems in clinical trials.

The overall aim of this section is to provide the investigator with a clear understanding of the possible risks and adverse reactions, and of the specific tests, observations, and precautions that may be needed for a clinical trial. This understanding should be based on the available physical, chemical, pharmaceutical, pharmacological, toxicological, and clinical information on the investigational product(s). Guidance should also be provided to the clinical investigator on the recognition and treatment of possible overdose and adverse drug reactions that is based on previous human experience and on the pharmacology of the investigational product.

7.4 APPENDIX 1:

TITLE PAGE *(Example)*

SPONSOR'S NAME

Product:
Research Number:
Name(s): Chemical, Generic (if approved)
Trade Name(s) (if legally permissible and desired by the sponsor)

INVESTIGATOR'S BROCHURE

Edition Number:
Release Date:

Replaces Previous Edition Number:
Date:

7.5 APPENDIX 2:

TABLE OF CONTENTS OF INVESTIGATOR'S BROCHURE *(Example)*

- Confidentiality Statement (optional) ....
- Signature Page (optional) ....
1 Table of Contents ....
2 Summary ....
3 Introduction ....
4 Physical, Chemical, and Pharmaceutical Properties and Formulation ....
5 Nonclinical Studies ....
5.1 Nonclinical Pharmacology ....
5.2 Pharmacokinetics and Product Metabolism in Animals ....
5.3 Toxicology ....
6 Effects in Humans ....
6.1 Pharmacokinetics and Product Metabolism in Humans ....
6.2 Safety and Efficacy ....
6.3 Marketing Experience ....
7 Summary of Data and Guidance for the Investigator ....

NB: References on 1. Publications
2. Reports

These references should be found at the end of each chapter

Appendices (if any)

8. ESSENTIAL DOCUMENTS FOR THE CONDUCT OF A CLINICAL TRIAL

8.1 Introduction

Essential Documents are those documents which individually and collectively permit evaluation of the conduct of a trial and the quality of the data produced. These documents serve to demonstrate the compliance of the investigator, sponsor and monitor with the standards of Good Clinical Practice and with all applicable regulatory requirements.

Essential Documents also serve a number of other important purposes. Filing essential documents at the investigator/institution and sponsor sites in a timely manner can greatly assist in the successful management of a trial by the investigator, sponsor and monitor. These documents are also the ones which are usually audited by the sponsor's independent audit function and inspected by the regulatory authority(ies) as part of the process to confirm the validity of the trial conduct and the integrity of data collected.

The minimum list of essential documents which has been developed follows. The various documents are grouped in three sections according to the stage of the trial during which they will normally be generated: 1) before the clinical phase of the trial commences, 2) during the clinical conduct of the trial, and 3) after completion or termination of the trial. A description is given of the purpose of each document, and whether it should be filed in either the investigator/institution or sponsor files, or both. It is acceptable to combine some of the documents, provided the individual elements are readily identifiable.

Trial master files should be established at the beginning of the trial, both at the investigator/institution's site and at the sponsor's office. A final close-out of a trial can only be done when the monitor has reviewed both investigator/institution and sponsor files and confirmed that all necessary documents are in the appropriate files.

Any or all of the documents addressed in this guideline may be subject to, and should be available for, audit by the sponsor's auditor and inspection by the regulatory authority(ies).

8.2 Before the Clinical Phase of the Trial Commences

During this planning stage the following documents should be generated and should be on file before the trial formally starts

	Title of Document	Purpose	Located in Files of Investigator/ Institution	Located in Files of Sponsor
**8.2.1**	**INVESTIGATOR'S BROCHURE**	To document that relevant and current scientific information about the investigational product has been provided to the investigator	X	X
**8.2.2**	**SIGNED PROTOCOL AND AMENDMENTS, IF ANY, AND SAMPLE CASE REPORT FORM (CRF)**	To document investigator and sponsor agreement to the protocol/amendment(s) and CRF	X	X
**8.2.3**	**INFORMATION GIVEN TO TRIAL SUBJECT**		X	X
	**- INFORMED CONSENT FORM** (including all applicable translations)	To document the informed consent		
	**- ANY OTHER WRITTEN INFORMATION**	To document that subjects will be given appropriate written information (content and wording) to support their ability to give fully informed consent	X	X
	**- ADVERTISEMENT FOR SUBJECT RECRUITMENT** (if used)	To document that recruitment measures are appropriate and not coercive	X	
**8.2.4**	**FINANCIAL ASPECTS OF THE TRIAL**	To document the financial agreement between the investigator/institution and the sponsor for the trial	X	X

	Title of Document	Purpose	Located in Files of Investigator/ Institution	Located in Files of Sponsor
8.2.5	**INSURANCE STATEMENT** (where required)	To document that compensation to subject(s) for trial-related injury will be available	X	X
8.2.6	**SIGNED AGREEMENT BETWEEN INVOLVED PARTIES,** e.g.:	To document agreements		
	- investigator/institution and sponsor		X	X
	- investigator/institution and CRO		X	X (where required)
	- sponsor and CRO			X
	- investigator/institution and authority(ies) (where required)		X	X
8.2.7	**DATED, DOCUMENTED APPROVAL/FAVOURABLE OPINION OF INSTITUTIONAL REVIEW BOARD (IRB) /INDEPENDENT ETHICS COMMITTEE (IEC) OF THE FOLLOWING:**<br>- protocol and any amendments<br>- CRF (if applicable)<br>- informed consent form(s)<br>- any other written information to be provided to the subject(s)<br>- advertisement for subject recruitment (if used)<br>- subject compensation (if any)<br>- any other documents given approval/ favourable opinion	To document that the trial has been subject to IRB/IEC review and given approval/favourable opinion. To identify the version number and date of the document(s)	X	X

	Title of Document	Purpose	Located in Files of Investigator/ Institution	Located in Files of Sponsor
**8.2.8**	**INSTITUTIONAL REVIEW BOARD/INDEPENDENT ETHICS COMMITTEE COMPOSITION**	To document that the IRB/IEC is constituted in agreement with GCP	X	X (where required)
**8.2.9**	**REGULATORY AUTHORITY(IES) AUTHORISATION/APPROVAL/ NOTIFICATION OF PROTOCOL** (where required)	To document appropriate authorisation/approval/notification by the regulatory authority(ies) has been obtained prior to initiation of the trial in compliance with the applicable regulatory requirement(s)	X (where required)	X (where required)
**8.2.10**	**CURRICULUM VITAE AND/OR OTHER RELEVANT DOCUMENTS EVIDENCING QUALIFICATIONS OF INVESTIGATOR(S) AND SUB-INVESTIGATOR(S)**	To document qualifications and eligibility to conduct trial and/or provide medical supervision of subjects	X	X
**8.2.11**	**NORMAL VALUE(S)/RANGE(S) FOR MEDICAL/ LABORATORY/TECHNICAL PROCEDURE(S) AND/OR TEST(S) INCLUDED IN THE PROTOCOL**	To document normal values and/or ranges of the tests	X	X
**8.2.12**	**MEDICAL/LABORATORY/TECHNICAL PROCEDURES /TESTS**<br>- certification or<br>- accreditation or<br>- established quality control and/or external quality assessment or<br>- other validation (where required)	To document competence of facility to perform required test(s) , and support reliability of results	X (where required)	X

	Title of Document	Purpose	Located in Files of Investigator/ Institution	Located in Files of Sponsor
8.2.13	**SAMPLE OF LABEL(S) ATTACHED TO INVESTIGATIONAL PRODUCT CONTAINER(S)**	To document compliance with applicable labelling regulations and appropriateness of instructions provided to the subjects		X
8.2.14	**INSTRUCTIONS FOR HANDLING OF INVESTIGATIONAL PRODUCT(S) AND TRIAL-RELATED MATERIALS** (if not included in protocol or Investigator's Brochure)	To document instructions needed to ensure proper storage, packaging, dispensing and disposition of investigational products and trial-related materials	X	X
8.2.15	**SHIPPING RECORDS FOR INVESTIGATIONAL PRODUCT(S) AND TRIAL-RELATED MATERIALS**	To document shipment dates, batch numbers and method of shipment of investigational product(s) and trial-related materials. Allows tracking of product batch, review of shipping conditions, and accountability	X	X
8.2.16	**CERTIFICATE(S) OF ANALYSIS OF INVESTIGATIONAL PRODUCT(S) SHIPPED**	To document identity, purity, and strength of investigational product(s) to be used in the trial		X
8.2.17	**DECODING PROCEDURES FOR BLINDED TRIALS**	To document how, in case of an emergency, identity of blinded investigational product can be revealed without breaking the blind for the remaining subjects' treatment	X	X (third party if applicable)

Title of Document		Purpose	Located in Files of	
			**Investigator/ Institution**	**Sponsor**
**8.2.18**	**MASTER RANDOMISATION LIST**	To document method for randomisation of trial population		X (third party if applicable)
**8.2.19**	**PRE-TRIAL MONITORING REPORT**	To document that the site is suitable for the trial (may be combined with 8.2.20)		X
**8.2.20**	**TRIAL INITIATION MONITORING REPORT**	To document that trial procedures were reviewed with the investigator and the investigator's trial staff (may be combined with 8.2.19)	X	X

8.3 During the Clinical Conduct of the Trial

In addition to having on file the above documents, the following should be added to the files during the trial as evidence that all new relevant information is documented as it becomes available

**8.3.1**	**INVESTIGATOR'S BROCHURE UPDATES**	To document that investigator is informed in a timely manner of relevant information as it becomes available	X	X

	Title of Document	Purpose	Located in Files of Investigator/ Institution	Located in Files of Sponsor
8.3.2	**ANY REVISION TO:**<br>- protocol/amendment(s) and CRF<br>- informed consent form<br>- any other written information provided to subjects<br>- advertisement for subject recruitment (if used)	To document revisions of these trial related documents that take effect during trial	X	X
8.3.3	**DATED, DOCUMENTED APPROVAL/FAVOURABLE OPINION OF INSTITUTIONAL REVIEW BOARD (IRB) /INDEPENDENT ETHICS COMMITTEE (IEC) OF THE FOLLOWING:**<br>- protocol amendment(s)<br>- revision(s) of:<br>- informed consent form<br>- any other written information to be provided to the subject<br>- advertisement for subject recruitment (if used)<br>- any other documents given approval/favourable opinion<br>- continuing review of trial (where required)	To document that the amendment(s) and/or revision(s) have been subject to IRB/IEC review and were given approval/favourable opinion. To identify the version number and date of the document(s).	X	X

	Title of Document	Purpose	Located in Files of Investigator/ Institution	Located in Files of Sponsor
**8.3.4**	**REGULATORY AUTHORITY(IES) AUTHORISATIONS/APPROVALS/NOTIFICATIONS WHERE REQUIRED FOR:**<br>- protocol amendment(s) and other documents	To document compliance with applicable regulatory requirements	X<br>(where required)	X
**8.3.5**	**CURRICULUM VITAE FOR NEW INVESTIGATOR(S) AND/OR SUB-INVESTIGATOR(S)**	(see 8.2.10)	X	X
**8.3.6**	**UPDATES TO NORMAL VALUE(S)/RANGE(S) FOR MEDICAL/ LABORATORY/ TECHNICAL PROCEDURE(S)/TEST(S) INCLUDED IN THE PROTOCOL**	To document normal values and ranges that are revised during the trial (see 8.2.11)	X	X
**8.3.7**	**UPDATES OF MEDICAL/LABORATORY/ TECHNICAL PROCEDURES/TESTS**<br>- certification or<br>- accreditation or<br>- established quality control and/or external quality assessment or<br>- other validation (where required)	To document that tests remain adequate throughout the trial period (see 8.2.12)	X<br>(where required)	X
**8.3.8**	**DOCUMENTATION OF INVESTIGATIONAL PRODUCT(S) AND TRIAL-RELATED MATERIALS SHIPMENT**	(see 8.2.15.)	X	X

	Title of Document	Purpose	Located in Files of Investigator/ Institution	Located in Files of Sponsor
**8.3.9**	**CERTIFICATE(S) OF ANALYSIS FOR NEW BATCHES OF INVESTIGATIONAL PRODUCTS**	(see 8.2.16)		X
**8.3.10**	**MONITORING VISIT REPORTS**	To document site visits by, and findings of, the monitor		X
**8.3.11**	**RELEVANT COMMUNICATIONS OTHER THAN SITE VISITS**<br>- letters<br>- meeting notes<br>- notes of telephone calls	To document any agreements or significant discussions regarding trial administration, protocol violations, trial conduct, adverse event (AE) reporting	X	X
**8.3.12**	**SIGNED INFORMED CONSENT FORMS**	To document that consent is obtained in accordance with GCP and protocol and dated prior to participation of each subject in trial. Also to document direct access permission (see 8.2.3)	X	
**8.3.13**	**SOURCE DOCUMENTS**	To document the existence of the subject and substantiate integrity of trial data collected. To include original documents related to the trial, to medical treatment, and history of subject	X	

	Title of Document	Purpose	Located in Files of Investigator/ Institution	Located in Files of Sponsor
**8.3.14**	**SIGNED, DATED AND COMPLETED CASE REPORT FORMS (CRF)**	To document that the investigator or authorised member of the investigator's staff confirms the observations recorded	X (copy)	X (original)
**8.3.15**	**DOCUMENTATION OF CRF CORRECTIONS**	To document all changes/additions or corrections made to CRF after initial data were recorded	X (copy)	X (original)
**8.3.16**	**NOTIFICATION BY ORIGINATING INVESTIGATOR TO SPONSOR OF SERIOUS ADVERSE EVENTS AND RELATED REPORTS**	Notification by originating investigator to sponsor of serious adverse events and related reports in accordance with 4.11	X	X
**8.3.17**	**NOTIFICATION BY SPONSOR AND/OR INVESTIGATOR, WHERE APPLICABLE, TO REGULATORY AUTHORITY(IES) AND IRB(S)/IEC(S) OF UNEXPECTED SERIOUS ADVERSE DRUG REACTIONS AND OF OTHER SAFETY INFORMATION**	Notification by sponsor and/or investigator, where applicable, to regulatory authorities and IRB(s)/IEC(s) of unexpected serious adverse drug reactions in accordance with 5.17 and 4.11.1 and of other safety information in accordance with 5.16.2 and 4.11.2	X (where required)	X
**8.3.18**	**NOTIFICATION BY SPONSOR TO INVESTIGATORS OF SAFETY INFORMATION**	Notification by sponsor to investigators of safety information in accordance with 5.16.2	X	X
**8.3.19**	**INTERIM OR ANNUAL REPORTS TO IRB/IEC AND AUTHORITY(IES)**	Interim or annual reports provided to IRB/IEC in accordance with 4.10 and to authority(ies) in accordance with 5.17.3	X	X (where required)

	Title of Document	Purpose	Located in Files of Investigator/ Institution	Located in Files of Sponsor
**8.3.20**	**SUBJECT SCREENING LOG**	To document identification of subjects who entered pre-trial screening	X	X (where required)
**8.3.21**	**SUBJECT IDENTIFICATION CODE LIST**	To document that investigator/institution keeps a confidential list of names of all subjects allocated to trial numbers on enrolling in the trial. Allows investigator/institution to reveal identity of any subject	X	
**8.3.22**	**SUBJECT ENROLMENT LOG**	To document chronological enrolment of subjects by trial number	X	
**8.3.23**	**INVESTIGATIONAL PRODUCTS ACCOUNTABILITY AT THE SITE**	To document that investigational product(s) have been used according to the protocol	X	X
**8.3.24**	**SIGNATURE SHEET**	To document signatures and initials of all persons authorised to make entries and/or corrections on CRFs	X	X
**8.3.25**	**RECORD OF RETAINED BODY FLUIDS/ TISSUE SAMPLES (IF ANY)**	To document location and identification of retained samples if assays need to be repeated	X	X

8.4 After Completion or Termination of the Trial

After completion or termination of the trial, all of the documents identified in sections 8.2 and 8.3 should be in the file together with the following

	Title of Document	Purpose	Located in Files of Investigator/ Institution	Located in Files of Sponsor
8.4.1	**INVESTIGATIONAL PRODUCT(S) ACCOUNTABILITY AT SITE**	To document that the investigational product(s) have been used according to the protocol. To documents the final accounting of investigational product(s) received at the site, dispensed to subjects, returned by the subjects, and returned to sponsor	X	X
8.4.2	**DOCUMENTATION OF INVESTIGATIONAL PRODUCT DESTRUCTION**	To document destruction of unused investigational products by sponsor or at site	X (if destroyed at site)	X
8.4.3	**COMPLETED SUBJECT IDENTIFICATION CODE LIST**	To permit identification of all subjects enrolled in the trial in case follow-up is required. List should be kept in a confidential manner and for agreed upon time	X	
8.4.4	**AUDIT CERTIFICATE** (if available)	To document that audit was performed		X
8.4.5	**FINAL TRIAL CLOSE-OUT MONITORING REPORT**	To document that all activities required for trial close-out are completed, and copies of essential documents are held in the appropriate files		X
8.4.6	**TREATMENT ALLOCATION AND DECODING DOCUMENTATION**	Returned to sponsor to document any decoding that may have occurred		X

	Title of Document	Purpose	Located in Files of Investigator/ Institution	Sponsor
**8.4.7**	**FINAL REPORT BY INVESTIGATOR TO IRB/IEC WHERE REQUIRED, AND WHERE APPLICABLE, TO THE REGULATORY AUTHORITY(IES)**	To document completion of the trial	X	
**8.4.8**	**CLINICAL STUDY REPORT**	To document results and interpretation of trial	X (if applicable)	X
